# Selective Uptake of Carboxylated Multi-Walled Carbon Nanotubes by Class A Type 1 Scavenger Receptors and Impaired Phagocytosis in Alveolar Macrophages

**DOI:** 10.3390/nano10122417

**Published:** 2020-12-03

**Authors:** Ruhung Wang, Rishabh Lohray, Erik Chow, Pratima Gangupantula, Loren Smith, Rockford Draper

**Affiliations:** 1 Department of Biological Sciences, The University of Texas at Dallas, 800 West Campbell Road, Richardson, TX 75080, USA; ruhung.wang@utdallas.edu (R.W.); Rishabh.Lohray@bcm.edu (R.L.); Pratima.Gangupantula@utdallas.edu (P.G.); 2 Department of Chemistry & Biochemistry, The University of Texas at Dallas, 800 West Campbell Road, Richardson, TX 75080, USA; Loren@utdallas.edu; 3 Department of Bioengineering, The University of Texas at Dallas, 800 West Campbell Road, Richardson, TX 75080, USA; ec829@cornell.edu

**Keywords:** nanomaterials, macrophages, class A type 1 scavenger receptors, cytotoxicity, macrophage–nanoparticle interaction

## Abstract

The production and applications of multi-walled carbon nanotubes (MWNTs) have increased despite evidence that MWNTs can be toxic. Recently, we reported that the binding of Pluronic^®^ F-108 (PF108)-coated carboxylated MWNTs (C-MWNTs) to macrophages is inhibited by class A scavenger receptors (SR-As) antagonists (R. Wang et al., 2018. Nanotoxicology 12:677–690). The current study investigates the uptake of PF108-coated MWNTs by macrophages lacking SR-A1 and by CHO cells that ectopically express SR-A1. Macrophages without SR-A1 failed to take up C-MWNTs and CHO cells that expressed SR-A1 did take up C-MWNTs, but not pristine MWNTs (P-MWNTs) or amino-functionalized MWNTs (N-MWNTs). The dependence of C-MWNT uptake on SR-A1 is strong evidence that SR-A1 is a receptor for C-MWNTs. The consequences of SR-A1-dependent C-MWNT accumulation on cell viability and phagocytic activity in macrophages were also studied. C-MWNTs were more toxic than P-MWNTs and N-MWNTs in cell proliferation and colony formation tests. C-MWNTs reduced surface SR-A1 levels in RAW 264.7 cells and impaired phagocytic uptake of three known SR-A1 ligands, polystyrene beads, heat-killed *E. coli*, and oxLDL. Altogether, results of this study confirmed that SR-A1 receptors are important for the selective uptake of PF108-coated C-MWNTs and that accumulation of the C-MWNTs impairs phagocytic activity and cell viability in macrophages.

## 1. Introduction

Carbon nanotubes (CNTs) are graphene sheets rolled into cylindrical tubes. Single-walled carbon nanotubes (SWNTs) contain a single graphene tube while multi-walled carbon nanotubes (MWNTs) contain multiple tubes concentrically nested inside each other. The light weight, strength, electrical and thermal conductivity of CNTs make them suited for applications in diverse fields including flexible electronics, medicine, reinforced composites, sensors, and Li-ion batteries [1,2,3]. The production of various CNT types is expected to exceed 15 kilotons/year by 2020 [3]. The increased production and use of CNTs raises the risk of unwanted exposure and subsequent toxicity. Sustained exposure has been shown to cause pulmonary inflammation [4,5], fibrosis [6], gene damage [7], and even mesothelioma [8] in lab animals. A better understanding of the pathology of CNT exposure is important to facilitate the design of less toxic CNT forms and to develop rational strategies for treating persons who may be accidentally exposed.

The surfaces of CNTs are often chemically functionalized, for example, by addition of carboxyl or amino groups, to tune their properties for increased dispersibility in aqueous solution, for decreased toxicity, or for higher drug load and targeting specificity in advanced drug delivery options. In nanomedicine, specific CNT functionalizations have been shown to facilitate the delivery of anticancer agents and biomolecules to target tissues for cancer therapy, thermal ablation therapy, gene therapy, immunotherapy, and for diagnostic applications [9]. However, these applications encounter a common significant challenge, where 30–99% of nanoparticles administered in vivo are sequestered by liver macrophages [10], and the mechanisms underlying the interaction between CNTs and macrophages remain poorly understood.

Recently, we reported that both human and mouse alveolar macrophages accumulated far more Pluronic^®^ F-108 (PF108)-coated carboxylated MWNTs (C-MWNTs) and carboxylated SWNTs (C-SWNTs) at 37 °C than the non-functionalized pristine MWNTs (P-MWNTs) and SWNTs (P-SWNTs) [11]. In addition, more C-MWNTs than P-MWNTs bound to macrophages in direct binding assays at 4 °C [11]. These data suggested that there were cell surface receptors that bound C-MWNTs but not P-MWNTs. Since the binding of C-MWNTs and C-SWNTs to macrophages was inhibited by known antagonists of class A scavenger receptors [11], members of this receptor class may be involved in the specific uptake of carboxylated CNTs in macrophages.

Class A scavenger receptors (SR-As) are pattern recognition receptors expressed by various cell types including macrophages, endothelial cells, and dendritic cells [12,13,14]. There are six types of SR-As, classified by a consensus nomenclature, which all contain a collagenous domain believed to bind a wide variety of polyanionic ligands, including modified low density lipoproteins, polysaccharides, nucleic acids, and various bacteria [14]. Since C-MWNTs are polyanions, it is possible that they could be SR-A ligands. Dextran sulfate and fucoidan are known antagonists of SR-A ligands, and in previous work we noted that both of these compounds partially blocked the binding of C-MWNTs to RAW 264.7 macrophages [11]. In addition, RAW 264.7 cells express high levels of scavenger receptor A1 (SR-A1) [15], leading to the hypothesis that SR-A1 might be a receptor for C-MWNTs. However, polyanionic inhibitors may affect more than one type of SR-A and it is difficult to pinpoint which type may be interacting with C-MWNTs. One main objective of the present paper was to test the hypothesis that SR-A1 is a receptor for C-MWNTs by measuring C-MWNT accumulation by macrophages that lack SR-A1 and by Chinese Hamster Ovary K1 cell (CHO-K1) clones that have been transfected with mouse SR-A1 cDNA. SR-A1 deficient macrophages and wild type CHO-K1 that do not normally express SR-A1 failed to accumulate significant amounts of C-MWNTs, whereas SR-A1 expressing CHO-K1 clones did accumulate C-MWNTs, strong evidence that SR-A1 is a C-MWNT receptor.

Possible physiological consequences of C-MWNT accumulation on cell viability and SR-A1 function were also explored in the current study. RAW 264.7 cell proliferation and colony formation efficiency were impaired in a dose and time dependent manner after exposure to C-MWNTs. In addition, exposure of RAW 264.7 cells to C-MWNTs reduced the level of SR-A1 on their surface and reduced the uptake of three known SR-A1 ligands: polystyrene beads, heat-killed *E. coli*, and oxidized low density lipoprotein (oxLDL).

## 2. Materials and Methods

### 2.1. MWNTs and other Materials

Three different research grade MWNT powders were used in this work, all purchased from NanoCyl (NanoCyl SA, Sambreville, Belgium). (1) Non-functionalized pristine MWNTs (P-MWNTs, NC3150^TM^); (2) Carboxyl-functionalized MWNTs (C-MWNTs, NC3151^TM^); and (3) Amino-functionalized MWNTs (N-MWNTs, NC3152^TM^). The MWNTs were produced by the catalytic chemical vapor deposition process to an average outside diameter of ~9.5 nm, purified to >95 wt.% carbon content and shortened to an average length of <1.0 µm, according to the product specifications provided by the manufacturer. Proprietary surface modification methods were used by the manufacturer to introduce <8.0 wt.% content of -COOH groups in the C-MWNT product and <0.6 wt.% of -NH_2_ groups in the N-MWNT product. Additional physicochemical properties of these and other MWNT products, including bulk metal catalysts composition by energy dispersive X-ray spectroscopy (EDX) and surface chemical compositions by X-ray photoelectron spectroscopy (XPS), are available from previous work in the literature [16,17]. Caution: a fine particulate respirator and other appropriate personal protective equipment should be worn when handling dry MWNT powders.

Pluronic^®^ F-108 (PF108) (cat. No. 542342), G418 disulfate salt solution (cat. No. G8168), IgG from mouse serum (cat. No. I5381), and Trypan Blue solution (cat. No. T8154) were purchased from Sigma Aldrich (St. Louis, MO, USA). Mouse SR-AI/MSR Alexa Fluor^®^ 488-conjugated antibody (cat. No. FAB1797G), rat IgG2b Alexa Fluor^®^ 488-conjugated isotype control antibody (cat. No. IC013G), mouse MARCO allophycocyanin (APC)-conjugated antibody (cat. No. FAB2956A), rat IgG1 APC-conjugated isotype control antibody (cat. No. IC005A), and flow cytometry (FCyt) staining buffer (cat. No. FC001) were purchased from R&D Systems, Inc. (Minneapolis, MN, USA) and used as received. Non-functionalized fluorescent polystyrene beads with a nominal diameter of 1 μm were acquired from Bangs Laboratories Inc. (cat. No. FSDG004, Fishers, Indiana). According to the manufacturer, the beads were produced by an emulsion polymerization technique resulting in a net negative surface charge. They were internally labeled with Dragon Green Dye (Ex: 480 nm, Em: 520 nm), using a solvent swelling/dye entrapment technique. Annexin V-FITC apoptosis detection kit (Invitrogen^TM^ cat. No. V13242), heat-killed Alexa Fluor^®^ 488 conjugated *Escherichia coli* (K-12 strain) BioParticles™ (Invitrogen^TM^ cat. No. E13231), and oxidized low density lipoprotein from human plasma (Invitrogen^TM^ cat. No. L34357) were purchased from Thermo Fisher Scientific (Waltham, MA, USA). Oxidized LDL uptake assay kit was purchased from Cayman Chemical (cat. No. 601180, Ann Arbor, MI, USA).

### 2.2. Preparation and Characterization of PF108 MWNT Dispersions

Pluronic^®^ F-108 (PF108) is a non-ionic triblock copolymer, also known as poloxamer 338. PF108 and related poloxamers have been used as effective surfactants to prepare aqueous dispersions of hydrophobic nanomaterials, including MWNTs and SWNTs, for nanotoxicity studies [11,18,19]. A stock PF108 solution at 5 mM concentration was prepared by dissolving PF108 powder in DI water purified using a Milli-Q system (Billerica, MA, USA), filtered through a 0.22 µm membrane, and stored at 4 °C in the dark. All MWNT dispersions were prepared with a freshly diluted and filtered 0.2 mM PF108 solution. To reduce potential endotoxin contaminants that could lead to ambiguous toxicity results, all MWNT powders were baked at 200 °C for 2 h [20] before PF108 solution was added. The sonication, centrifugation, and dialysis protocols described in our previous work [18,19] were used to prepare PF108-coated MWNT dispersions. Note that the dialysis step is crucial to remove toxic PF108 products generated by sonication [18,21]. The prepared dispersions of P-, N-, and C-MWNTs in PF108 solution were denoted as PMPF, NMPF, and CMPF dispersions, respectively.

The concentration of MWNTs in each prepared dispersion was measured using the absorbance at 500 nm. Dynamic light scattering (DLS) and zeta potential (ZP) analyses were used as part of a quality control routine for the preparation of all MWNT dispersions [18,19]. In addition, zeta potentials of all MWNT dispersions diluted to ~50 µg/mL in water and in cell culture medium with 10% fetal bovine serum (FBS) were acquired at 25 °C and 37 °C, respectively.

The physicochemical properties of the three MWNT powders provided by the manufacturer and the properties of PF108-coated MWNT dispersions prepared for this study are shown in Table 1. Note that the zeta potentials for C-MWNTs were slightly more negative than those for P- and N-MWNTs in water. Also, the zeta potential values were less negative for all MWNT types in medium with serum than in water, as expected due to the increase in salt and serum protein concentrations, and the C-MWNTs still had slightly more negative zeta potentials than P- or N-MWNTs.

### 2.3. Cell Lines and Cell Culture

Dulbecco’s modified Eagle medium (DMEM) was purchased from Gibco (Grand Island, NY, USA). Ham’s F-12K (ATCC^®^ 30-2004) and RPMI 1640 (ATCC^®^ 30-2001) media were purchased from the American Type Culture Collection (ATCC, Manassas, VA, USA). FBS was purchased from Atlanta Biologicals (Flowery Branch, GA, USA). Penicillin-streptomycin solution (100 U penicillin/0.1 mg streptomycin per mL) was purchased from Sigma Aldrich and used only in terminal cultures. Gibco^TM^ PBS-based enzyme-free cell dissociation buffer (cat. No. 13151-014), Accumax^TM^ (cat. No. 00-4666-56), Accutase^TM^ (cat. No. A1110501), and 10× concentrated phosphate buffered saline (cat. No. BP399-1) were purchased from Thermo Fisher Scientific (Waltham, MA, USA).

Abelson murine leukemia virus transformed macrophage RAW 264.7 cells (ATCC^®^ TIB-71) and Chinese Hamster Ovary CHO-K1 cells (ATCC^®^ CCL-61) were purchased from ATCC. Two immortalized alveolar macrophage cell lines, B6 and ZK, were kindly provided by Prof. L. Kobzik (retired, Harvard TH Chan School of Public Health, Boston, MA, USA). B6 cells were derived from wild type (WT) C57BL/6 mice and ZK cells were derived from MARCO and SR-AI/II deficient (MS^−/−^) mice [22]. RAW 264.7 cells were cultured in DMEM base medium and WT B6 and MS^−/−^ ZK cells were cultured in RPMI 1640 base medium. CHO cells stably transfected with full length mouse SR-A1 cDNA, termed CHO[mSR-AI] cells [23], were kindly provided by Prof. M. Krieger (Massachusetts Institute of Technology, Boston, MA, USA). As received CHO[mSR-AI] cells were cultured in selective medium containing 0.5 mg/mL of geneticin (G418) and single colonies were isolated by dilution plating. The surviving colonies were screened for high surface SR-A1 receptor expression by immuno-fluorescent FCyt, from which three sub-clones, termed CHO + mSRA1.A, CHO + mSRA1.B, and CHO + mSRA1.C were selected. CHO-K1 and transfected CHO + mSRA1.A, CHO + mSRA1.B, and CHO + mSRA1.C cells were cultured in F-12K base medium. All regular culture media were supplemented with 1.5 mg/mL sodium bicarbonate, 10% (*v*/*v*) FBS, and 10 mM HEPES buffer at pH 7.4 for cells cultured in a 37 °C incubator with 95% air and 5% CO_2_. For cells incubated in a 4 °C incubator without CO_2_ supplement, no sodium bicarbonate was added to the media to maintain proper pH.

### 2.4. Surface Expression of Class A Type 1 Scavenger Receptors SR-A1 and MARCO

Macrophage cell surface expression of SR-A1 and MARCO receptors was determined by a direct immunofluorescence FCyt assay. Macrophages cultured in 6-well plates at 37 °C were washed three times with warm PBS to remove serum components present in the media and detached from the plate with gentle pipetting in enzyme-free PBS. Cells in suspension were washed twice with cold PBS and aliquots of ~1 × 10^6^ cells/100 µL in cold PBS were prepared. Macrophages often express Fc receptors on their surface that can bind to the Fc portion of a fluorescent reporter antibody. To block Fc receptors, 10 µg of unlabeled mouse IgG was added and incubated with the cells for 15 min at 4 °C. For immunostaining of surface SR-A1, 1 µg of rat anti-mouse mSR-AI/MSR Alexa Fluor^®^ 488-conjugated monoclonal antibody (R&D Systems cat. No. FAB1797G) was added and incubated with the cells for 30 min at 4 °C in the dark. A duplicate cell aliquot was incubated with an isotype control rat IgG2b Alexa Fluor^®^ 488-conjugated monoclonal antibody (R&D Systems cat. No. IC013G) to assess possible off-target staining by the anti-SR-A1 antibody. For immunostaining of surface MARCO receptors, a similar procedure was used but with a rat anti-mouse MARCO APC-conjugated monoclonal antibody (R&D Systems cat. No. FAB2956A) and the corresponding APC-conjugated rat IgG1 isotype control antibody (R&D Systems cat. No. IC005A). After incubation, unbound antibodies were washed away by centrifugation twice with 1 mL of cold FCyt staining buffer (R&D Systems cat. No. FC001) at 1000 × *g* for 8 min. The washed cells were re-suspended in cold buffer and stored on ice in the dark. Flow cytometric analysis of 20,000 counts per sample was used to determine the presence or absence of specific fluorescent antibodies bound to SR-A1 or MARCO receptors on the cell surface. Cells with fluorescence intensity greater than the background isotype control were considered positive for the receptors.

### 2.5. Accumulation of MWNTs in RAW 264.7, B6, ZK Macrophages and CHO Cell Lines

The sodium dodecyl sulfate polyacrylamide gel electrophoresis (SDS-PAGE) procedure described in our previous work [11,18,19] was used to determine the amount of MWNTs accumulated by cells after a 24 h exposure at 37 °C. Briefly, cells were seeded in 6-well plates in regular culture media overnight before incubating in either control media that contained no MWNTs or test media with 100 µg/mL of P-, N-, or C-MWNTs for 24 h at 37 °C. After incubation, cells were washed extensively with fresh medium and PBS and detached from culture plates with Accutase^TM^ cell dissociation buffer. Cell numbers were determined using a Coulter Particle Counter, and MWNTs were extracted from cells and quantified using the SDS-PAGE method. The average amount of P-, N-, or C-MWNTs accumulated by the cell over a 24 h period was expressed as fg MWNT per cell.

### 2.6. Apoptosis Assay

The induction of apoptosis in RAW 264.7 cells was assessed after a 24 h incubation with media containing either 0.1 mM PF108 alone, or 100 µg/mL of P-, N-, or C-MWNTs using an apoptosis detection kit (Invitrogen™ cat. No. V13242) with FITC-conjugated annexin V and propidium iodide (PI) duo fluorescent markers for FCyt analysis. A total of 3 × 10^4^ RAW 264.7 cells/well were seeded in 24-well plates and incubated at 37 °C overnight before the regular culture medium was replaced with freshly prepared control or test media and incubated for 24 h. Untreated cells provided a negative control and cells treated with 100 nM camptothecin (CPT) were used as a positive control for apoptosis. After the incubation, cells were washed 3 times with fresh medium and twice with PBS, then detached from culture plate by incubating in a PBS-based enzyme-free buffer for 5 min at 37 °C. Cells in suspension were washed, kept on ice, and stained with FITC-conjugated annexin V and PI for 15 min in the dark, according to the protocol provided by the kit. The externalization of phosphatidylserine in apoptotic cells was detected using green fluorescent-conjugated recombinant annexin V and the nuclei of dead or necrotic cells were detected using red fluorescent PI. After treatment with annexin V and PI, 10,000 cell counts per sample were analyzed using an FCyt cell analyzer (BD Accuri^TM^ C6 Plus flow cytometer) where binding of annexin V was recorded in the green fluorescent channel and the binding of PI in the red fluorescent channel. Data were presented as dot plots, where green fluorescence for annexin V-FITC was plotted on the *X*-axis with a threshold set to 2 × 10^4^, and red fluorescence for PI was plotted on the *Y*-axis with a threshold set to 1 × 10^4^. In general, apoptotic cells show higher green fluorescence, necrotic cells show both higher green and higher red fluorescent signals, dead cells show high red but not green fluorescence, and viable cells show little or no fluorescence higher than background levels. The fractions of viable, apoptotic, necrotic, and dead cells in 10,000 counts analyzed per measurement were recorded.

### 2.7. Crystal Violet Cell Proliferation Assay

A standardized cytotoxicity assay based on cell proliferation described previously in our MWNT toxicity work [11,18] was used for cytotoxicity assessments with RAW 264.7 cells exposed to various MWNT types, doses, and exposure times. Briefly, 4 × 10^4^ RAW 264.7 cells/well were seeded in 48-well plates and incubated at 37 °C overnight before the regular cell culture media was replaced with freshly prepared control or test media for a 24 h exposure. 1 × 10^4^ and 5 × 10^3^ cells/well were seeded for longer exposures of 48 h and 72 h, respectively. At the end of the incubation, cells were washed 3 times with fresh media, 2 times with PBS, and air-dried to fix the cells on the plate. Cell proliferation was determined using a crystal violet assay, as described in our previous work where it was also demonstrated that cell-associated CNTs do not interfere with the assay [24]. The proliferation of the untreated control cells was set to 100%. IC50 values were estimated from a linear regression dose-effect trend line where the concentration of MWNT is needed to inhibit cell proliferation by 50%.

### 2.8. Colony Formation Efficiency (CFE) Assay

A well-established CFE assay based on the ability of a cell to grow into a colony under the test condition [25] was used to assess the viability of RAW 264.7 cells exposed to different MWNT types and doses continuously for 8 day. 300 RAW 264.7 cells per well were seeded in 6-well plates, filled with 5 mL/well of control regular culture medium or test media that contained either P-, N-, or C-MWNTs at a final MWNTs concentration of 25, 50, or 100 µg/mL, and incubated at 37 °C without disturbance to allow colony formation. At the end of the 8 day incubation, colonies were washed gently twice with fresh medium and twice with PBS, fixed to the plate by air-drying at room temperature, and stained with crystal violet (0.1% *w*/*v* in 10% ethanol) for 15 min at room temperature. Excess unbound dye was removed by rinsing the plate with tap water gently. Images of the wells with stained colonies were acquired using a stereomicroscope (NikonSMZ745T with Nikon DS-Fi2 camera) with a 0.5× objective lens magnification. The number of colonies in a well was counted and colony formation efficiency (CFE) was defined as the ratio of the number of colonies formed over the number of cells seeded in the well. The CFE of the untreated control was set to 100% and the IC50 value was estimated from a linear regression dose-effect trend line where the concentration of MWNT is needed to inhibit CFE by 50%.

### 2.9. Detection of Surface SR-A1 on RAW 264.7 Cells by Laser Scanning Confocal Fluorescence Microscopy (LSCFM)

A total of 2 × 10^4^ RAW 264.7 cells/well were seeded on glass coverslips in 4-well plates in regular culture media supplemented with 10% FBS for 24 h at 37 °C. The culture medium was replaced with freshly prepared control medium that contained no MWNTs or test media containing 100 µg/mL of P-, N-, or C-MWNTs and incubated for 24 h at 37 °C. After incubation, control and test media were removed, cells were washed extensively, and chilled to 4 °C in media that contained no sodium bicarbonate. To block surface Fc receptors, 10 µg of mouse IgG was added and incubated with the cells for 15 min at 4 °C. For immunostaining of surface SR-A1, 1 µg of rat anti-mouse mSR-AI/MSR Alexa Fluor^®^ 488-conjugated monoclonal antibody (R&D Systems cat. No. FAB1797G) was added and incubated with the cells for 30 min at 4 °C in the dark. After incubation, cells were washed to remove unbound antibodies and fixed with 4% *w*/*v* paraformaldehyde in PBS at room temperature for 15 min. The nuclei were stained with Hoechst 33342 in PBS at room temperature for 5 min in the dark. The cells were then washed with PBS twice to remove excess Hoechst dye, rinsed in Milli-Q water, and the coverslips were mounted on glass slides in Fluoromount G.

Confocal fluorescence images were acquired at the Imaging and Histology Core Facility at UT Dallas using LSCFM (Olympus FV3000RS) with a 100× magnification UPLSAPO objective lens (NA 1.35) immersed in silicone oil. Blue fluorescence in the images denotes nuclei stained with Hoechst dye and was detected with Ex. 405 nm and Em. 430–470 nm wavelengths. Green fluorescence denoting surface SR-A1 was detected with Ex. 488 nm and Em. 500–600 nm wavelengths. At least 30 confocal stacks were acquired per field and Z-projected images were overlaid using *ImageJ* software. 3D rendering of cells was reconstructed from ~30 confocal images along the *Z*-axis and 360° rotating images were recorded as video clips using *ImageJ* software.

### 2.10. Phagocytosis of Polystyrene Beads Assessed by LSCFM, FCyt, and LSCRM

A total of 2 × 10^4^ cells/well were seeded in 4-well plates for FCyt analysis in regular culture media (DMEM for RAW 264.7 cells and RPMI 1640 for B6 and ZK cells) supplemented with 10% FBS for 24 h at 37 °C. For LSCFM analysis, glass coverslips were inserted in the wells before 2 × 10^4^ cells/well were seeded. The culture medium was replaced with either freshly prepared test media containing 100 µg/mL of P-, N-, or C-MWNTs or control medium containing no MWNTs and incubated for 20 h at 37 °C. After incubation, control and test media were removed and cells were washed extensively with fresh medium and followed by a 1 h chase period in fresh culture medium at 37 °C. The medium was then replaced with fresh medium containing fluorescent polystyrene beads (10 µg/mL for RAW 264.7 cells and 25 µg/mL for the B6 or ZK cells). The cells were exposed to the beads for 2 h at 37 °C. After the 2 h exposure, cells were washed extensively to remove excess beads in the media and chased in fresh medium at 37 °C for phagocytosis of surface-bound but not yet internalized beads.

For LSCFM analysis, cells on coverslips were washed extensively, fixed with 4% *w*/*v* paraformaldehyde in PBS at room temperature for 15 min, and washed twice with PBS. The nuclei were stained with Hoechst 33342 in PBS at room temperature for 5 min in the dark. The cells were washed with PBS twice to remove excess Hoechst dye, rinsed in Milli-Q water, and the coverslips were mounted on glass slides in Fluoromount G. The acquisition and processing of confocal fluorescence images of the cells were the same as those described in the previous section.

For FCyt analysis, cells were washed, detached from the well with Accumax^TM^, and re-suspended in PBS. The cells in suspension were washed again with PBS and kept on ice in the dark. 10,000–20,000 cells per sample were analyzed using a flow cytometer where the green fluorescence intensity correlates to the number of phagocytosed beads in a cell. Cells with a green fluorescence intensity greater than the background auto-fluorescence intensity are considered positive for phagocytosed beads. The mean fluorescence index (MFI) of a sample, obtained by multiplying the % of positive cells with phagocytosed beads and the mean fluorescence intensity, represents the phagocytic activity of the cells to take up polystyrene beads under the experimental conditions.

For laser scanning confocal Raman microscopy (LSCRM) analysis, RAW 264.7 cells were cultured on glass coverslips, incubated in control culture medium or in test medium containing 100 µg/mL C-MWNT for 2 h at 37 °C, washed extensively, chased in fresh culture for 30 min, prior to exposure to 25 µg/mL of non-fluorescence and non-functionalized polystyrene beads for 2 h at 37 °C, washed again, then air-dried to prepare for Raman microscopy scanning. The polystyrene beads and C-MWNTs phagocytosed by the control or C-MWNT treated cells were detected using a WITec 500R LSCRM system and the acquired Raman scan images were processed using *WITec Project 4 plus* software (see further details in the SI Methods).

### 2.11. Phagocytosis of Heat-Killed Fluorescent Bacteria Assessed by FCyt

A total of 4 × 10^5^ RAW 264.7 cells/well were seeded in 6-well plates in regular culture media supplemented with 10% FBS for 24 h at 37 °C. The culture medium was replaced with freshly prepared test media containing either 0.1 mM PF108 alone, 100 µg/mL of P-, N-, or C-MWNTs, or control medium that contained no PF108 or MWNTs and incubated for 24 h at 37 °C. After incubation, control and test media were removed and cells were washed extensively with fresh medium and followed by a 1-h chase period in fresh culture medium at 37 °C. Cells were chilled to 4 °C and exposed to heat killed, Alexa Fluor^®^ 488-conjugated *E. coli* particles, at 30 *E. coli* particles per cell in fresh cold medium for 1 h at 4 °C in the dark. After the 1h exposure, cells were washed extensively to remove unbound *E. coli*, chased in fresh medium at either 37 °C or 4 °C for 1 h, washed, and detached from the plate in enzyme-free buffer. Cells in suspension were washed twice in cold PBS and 20,000 cell counts per measurement were analyzed using a flow cytometer where the green fluorescence intensity correlates to the number of *E. coli* associated with a cell. Cells with a fluorescence intensity greater than the background auto-fluorescence intensity are considered positive for phagocytosed (for cells chased at 37 °C) or surface-bound (for cells kept at 4 °C) *E. coli*.

### 2.12. Distinguishing Extracellular from Internalized Florescent Markers by Trypan Blue Quenching

A common fluorescence quenching technique with trypan blue (TB) [26,27] was used to distinguish internalized fluorescent-labeled particles from those attached to the cell surface, such as the Alexa Fluor^®^ 488-conjugated monoclonal antibody specific for SR-A1 or the heat killed *E. coli* particles used in the current study. Since trypan blue dye does not penetrate cell membranes, it may quench the green fluorescence of extracellular, i.e., free and surface-bound fluorescent-labeled particles, but has no effect on the fluorescence of particles internalized by the cell. To quantify the extent of fluorescence quenching by trypan blue, flow cytometric measurements of a sample were acquired as usual, in the absence of trypan blue, followed immediately by consecutive analysis in the presence of 0.1% trypan blue dye. The fluorescence intensity quenched by trypan blue is defined as the difference in fluorescence intensities measured in the absence (−TB) and presence (+TB) of the dye. The percent quenching by trypan blue was calculated as [(−TB)−(+TB)]/(−TB), where the fluorescence intensity in the absence of dye was set to 100%. A sample with high trypan blue quenching implies that the fluorescent markers reside on the cell surface. On the contrary, a sample with no fluorescence quenching by trypan blue suggests that the fluorescent markers were internalized by the cells.

### 2.13. Uptake of Fluorescent and Non-Fluorescent OxLDL Assessed by FM, FCyt and Oil Red O (ORO) Stain

A total of 2 × 10^4^ cells/well were seeded in 4-well plates for FCyt analysis in regular culture media supplemented with 10% FBS for 24 h at 37 °C. For FM and ORO stain analysis, glass coverslips were inserted in the wells before cells were seeded. The culture medium was replaced with freshly prepared test media containing 0.1 mM PF108 alone, 100 µg/mL of P-, N-, or C-MWNTs, or with fresh control medium containing no PF108 nor MWNTs and incubated for 20–24 h at 37 °C. After incubation, control and test media were removed and cells were washed extensively with fresh serum-free medium supplemented with 3% *w*/*v* BSA (SF + BSA medium) and followed by a 4 h serum starvation period at 37 °C.

For fluorescent oxLDL uptake assays, cells were incubated with fresh serum free (SF) + BSA medium containing DyLight^TM^ 488-conjugated oxLDL (1:20 dilution for FM and 1:40 dilution for FCyt) for 2 h at 37 °C. Cells were then washed extensively to remove excess oxLDL in the media. For FM analysis, cells on coverslips were fixed with 4% *w*/*v* paraformaldehyde in PBS at room temperature for 15 min, washed again with PBS, and the nuclei were stained with Hoechst 33342 in PBS at room temperature for 5 min in the dark before the coverslips were mounted on glass slides in Fluoromount G. Epi-fluorescence images were acquired using an inverted fluorescence microscope (Nikon Eclipse TE2000) with a 60× magnification oil immersion objective lens (NA 1.40). Blue color in the images denotes nuclei stained with Hoechst 33342 dye, detected with Ex. 365 nm and Em. 435–485 nm wavelengths. Green fluorescent oxLDL was detected with Ex. 475 nm and Em. 500–550 nm wavelengths. The Nikon *NIS-Elements AR* (v.4.40.00) software was used for image acquisition and includes a 2D deconvolution module used to reduce the imperfection of convolution on the fluorescence cell images. For FCyt analysis, cells were washed with PBS, detached from the well with Accumax^TM^, washed again, and kept on ice in the dark. A total of 10,000 cells per sample were analyzed using a flow cytometer where the green fluorescence intensity correlates to the abundance of oxLDL in a cell. Cells with a green fluorescence intensity greater than the background auto-fluorescence intensity are considered positive for active oxLDL uptake. The mean fluorescence intensity of a sample represents the phagocytic activity of the cells to take up oxLDL under the experimental conditions.

To measure the uptake of non-fluorescent oxLDL using ORO staining, cells were incubated with fresh SF+BSA media with or without 25 µg/mL oxLDL for 16 h at 37 °C. After the incubation, cells were washed extensively and fixed with 4% *w*/*v* paraformaldehyde in PBS at room temperature for 15 min. The oil droplets present in cells were stained with a freshly prepared 0.3% *w*/*v* ORO solution in 60% isopropanol for 15 min at room temperature. The ORO working solution was prepared by diluting a stock 0.5% *w*/*v* ORO in isopropanol solution (Sigma, cat. No. O1391) with MilliQ H_2_O at a 3:2 ratio. The solution was mixed, set for 10 min, passed through a 0.22 µm PVDF membrane syringe filter unit (MillexGV by Millipore, cat. No. SLGV033RS), and used within 30 min. After staining with ORO, cells were washed extensively with water. To remove ORO stains on the glass coverslips and on the wells of culture plates, coverslips were removed from the wells carefully and dipped first in a beaker filled with clean water for 10 s, then dipped in a second beaker filled with 50% isopropanol for 10 s, quickly dipped in a third beaker filled with clean water for 10 s, and transferred to wells filled with PBS in new 4-well plates. Bright-field images were acquired using a digital inverted microscope cell imaging system (EVOS FL AMEFC-4300) with a 40× magnification objective lens (NA 0.65), illuminated with an LED transmitted light at 60% intensity, and saved as 24-bit color TIFF files. To quantitate the amount of ORO associated with cells, the dye was eluted in 200 µL/well of 100% isopropanol (Sigma, cat. No. C-2432). The plates were placed on an orbital shaker for 15 min at room temperature and 150 µL eluate from each well was transferred to a well in a 96-well plate. The absorbance at 510 nm, corresponding to the ORO absorbance peak, was measured using a BioTek Synergy 2 Multi-Mode microplate reader (Winooski, VT, USA).

## 3. Results

### 3.1. Surface SR-A1 and MARCO Receptor Expression in RAW 264.7, B6, and ZK Cells

**Figure 1:**
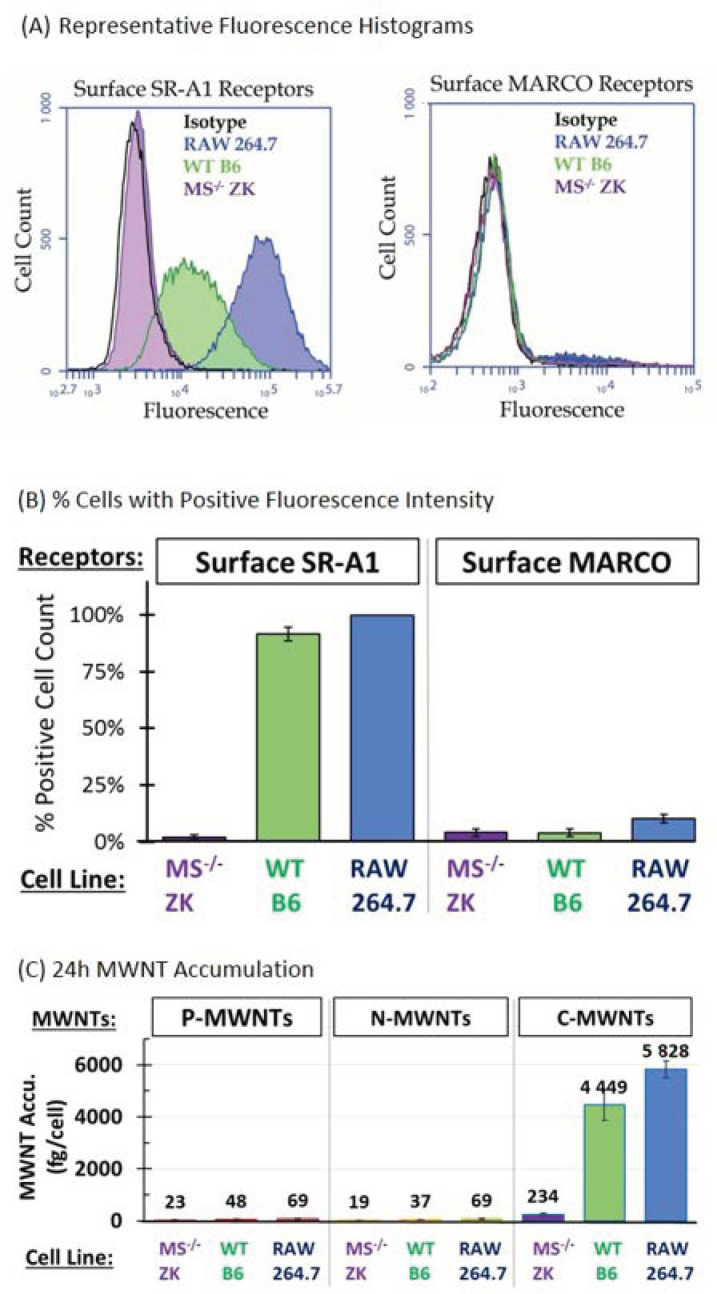
Surface SR-A1 receptor expression correlates with the accumulation of C-MWNTs by RAW 264.7, wild type (WT) B6, and MARCO and SR-AI/II deficient (MS)^−/−^ ZK murine alveolar macrophages. (**A**,**B**) Cell surface expression of SR-A1 and MARCO receptors was determined by direct immunofluorescence FCyt assays. Cells were prepared for cytometry as described in Methods using Alexa Fluor^®^ 488-conjugated monoclonal anti-mouse SR-A1 or APC-conjugated monoclonal anti-mouse MARCO antibody. Cells with fluorescence intensity greater than the background isotype control were considered positive. (**A**) Representative fluorescence histograms of cells stained with anti-mouse SR-A1 (left panel) or with anti-MARCO (right panel), and corresponding isotype control antibodies. Black, isotype control; blue, RAW 264.7 cells; green, WT B6 cells; purple, MS^−/−^ ZK cells. (**B**) The fraction of positive cells out of a total 20,000 analyzed was expressed as the % of cells positive for surface SR-A1 (left panel) or for surface MARCO (right panel) receptors. (**C**) The accumulation of P-, N-, or C-MWNTs by RAW 264.7, WT B6, and MS^−/−^ ZK cells was determined after exposing cells to media containing 100 µg/mL of either P-, N-, or C-MWNT for 24 h at 37 °C as described in Methods. Numbers above the bars are the average fg MWNTs per cell. Data in all panels of this figure are the average ± SD of ≥3 independent experiments.

The mouse alveolar macrophage-derived cell line RAW 264.7 and two immortalized alveolar macrophage cell lines, B6 and ZK, were studied to evaluate the participation of SR-A receptors in MWNT uptake. B6 cells are derived from wild type (WT) C57BL/6 mice and ZK cells are from MARCO and SR-AI/II deficient (MS^−/−^) mice [22]. The expression of surface SR-A1 and MARCO (SR-A6) receptors on RAW 264.7, WT B6, and MS^−/−^ ZK cells was detected by immunofluorescence staining and determined quantitatively using FCyt, as described in the Methods section. Representative histograms of surface SR-A1 and MARCO receptor expression with RAW 264.7, B6, and ZK cells are shown in Figure 1A. Fluorescent staining with respective isotype control mAbs was included as a negative control. Both RAW 264.7 and WT B6 cells express high levels of surface SR-A1 whereas MS^−/−^ ZK cells do not (Figure 1A, left panel). No relevant amount of MARCO receptors were detected on the surface of RAW 264.7, WT B6, or MS^−/−^ ZK cells (Figure 1A, right panel). For quantitative analysis, cells with fluorescence intensity greater than that of the isotype control are considered positive and the fraction of positive cells among 20,000 counts analyzed for each sample are plotted in Figure 1B. The results demonstrate that more than 90% of RAW 264.7 and WT B6 cells express surface SR-A1 and only 2% MS^−/−^ ZK cells tested positive for surface SR-A1. The MFI is the average fluorescence signal from the cells analyzed and gives a quantitative comparison of the population fluorescence. The MFI for RAW 264.7 and WT B6 cells was (102.4 ± 4.2) × 10^3^ and (22.4 ± 8.6) × 10^3^, respectively. In contrast, MS^−/−^ ZK cells express low surface SR-A1, evidenced by an MFI of (1.6 ± 1.5) × 10^3^, barely exceeding the isotype control background level MFI of (1.3 ± 0.6) × 10^3^. Unlike the SR-A1, the constitutive expression of the MARCO receptor appears to be low in all three alveolar macrophage cell lines tested (Figure 1B), with MFIs of (1.2 ± 0.7) × 10^3^, (0.8 ± 0.3) × 10^3^, and (0.7 ± 0.3) × 10^3^ for RAW 264.7, WT B6, and MS^−/−^ ZK mouse macrophages, respectively. This is consistent with prior studies indicating that RAW 264.7 cells express high levels of SR-A1 but not MARCO [15]. Note that the surface SR-A1 and MARCO receptor levels measured here using FCyt agree with PCR genotyping results reported previously by the Kobzik group [22]. Next, the amount of MWNTs accumulated by these cells was correlated with their expression of surface SR-A1 receptors.

### 3.2. Accumulation of MWNTs by RAW 264.7, WT B6, and MS^−/−^ ZK cells

Results of our previous study on MWNT uptake by RAW 264.7 cells suggested that class A scavenger receptors might be involved in the 100-fold preferential uptake of C-MWNTs over P-MWNTs [11]. In the present study, in addition to P- and C-MWNTs, the interaction of N-MWNTs with macrophages was assessed to see if a surface functionalization other than oxidation affected uptake by cells. After a 24 h incubation in media containing 100 µg/mL of PF108-coated MWNTs, RAW 264.7 cells accumulated 69 ± 18 and 69 ± 22 fg/cell of P- and N-MWNTs, respectively, and accumulated ~85 fold more C-MWNTs at 5828 ± 325 fg/cell (Figure 1C). This is consistent with our previous results [11] and shows that N-MWNTs, like P-MWNTs, are not highly accumulated. Under the same experimental conditions as with RAW 264.7 cells, WT B6 cells also accumulated ~100 fold more C-MWNTs than P- or N-MWNTs, with an average of 48 ± 25, 37 ± 8, and 4,449 ± 591 fg/cell of P-, N-, and C-MWNTs, respectively (Figure 1C). Notably, MS^−/−^ ZK cells that lack SR-A1 accumulated minimal P-, N-, or C-MWNTs (Figure 1C). Thus, high C-MWNT accumulation correlates with high expression of surface SR-A1. Moreover, since RAW 264.7 and WT B6 cells express minimal MARCO receptors on their surface (Figure 1A,B), MARCO receptors apparently cannot be responsible for the high C-MWNTs uptake in these cells (Figure 1C). To further investigate the effect of SR-A1 on the accumulation of MWNTs, transfected CHO cells that stably express mouse SR-A1 were studied next.

### 3.3. Selective High Uptake of C-MWNTs in CHO Cells Expressing SR-A1

CHO cells stably transfected with full length mouse class A type I scavenger receptor cDNA, termed CHO[mSR-AI] cells, were kindly provided by Prof. M. Krieger [23]. As received CHO[mSR-AI] cells were re-selected in medium containing 0.5 mg/mL of geneticin (G418, Gibco) and single colonies were isolated by dilution plating. The surviving colonies were screened for high surface SR-A1 expression by immunofluorescence FCyt, from which three sub-clones, termed CHO + mSRA1.A, CHO + mSRA1.B, and CHO + mSRA1.C, were selected and used to investigate the involvement of SR-A1 on the accumulation of P-, N-, and C-MWNTs by CHO cells.

**Figure 2:**
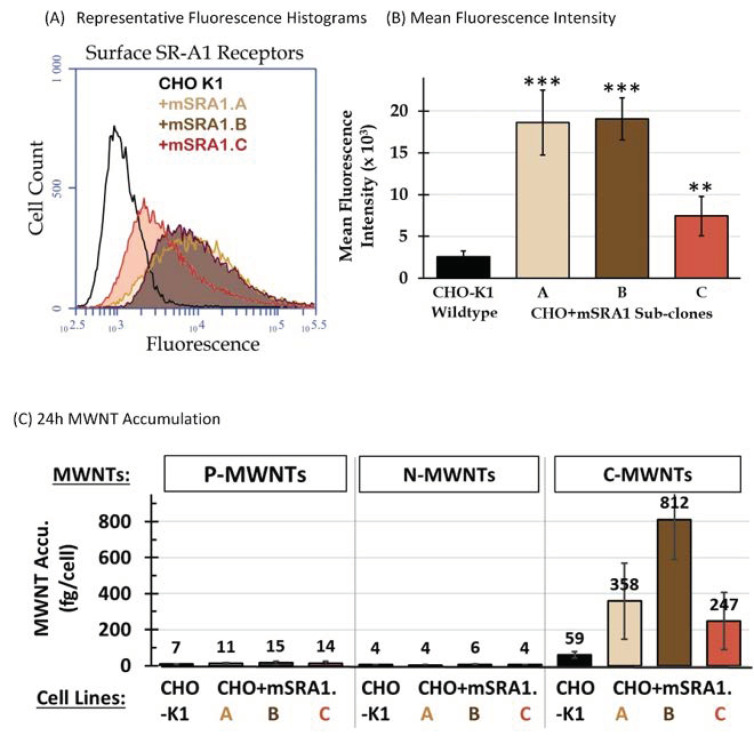
Surface SR-A1 receptor expression correlates with C-MWNT uptake by CHO-K1 and transfected CHO + mSRA1 clones. (**A**,**B**) Cell surface expression of SR-A1 receptors was determined by direct immunofluorescence FCyt assays. Cells detached from the culture vessel with enzyme-free buffer were incubated with mouse IgG to block Fc receptors before incubation with Alexa Fluor^®^ 488-conjugated monoclonal anti-mouse mSR-A1 antibody. Alexa Fluor^®^ 488-conjugated rat IgG2b was used as isotype control. Cells with fluorescence intensity greater than the background isotype control were considered positive. The mean fluorescence intensity represents the surface expression level of SR-A1 receptors by the 20,000 cells analyzed. (**A**) Representative fluorescence histograms of cells stained with anti-mSR-A1. Black, untransfected CHO-K1 cells; beige, brown, and red, transfected CHO + mSRA1 sub-clones A, B, and C, respectively. (**B**) The surface SR-A1 receptor expression level represented as the mean fluorescence intensity of 20,000 cells analyzed per sample. Data are the mean ± SD of triplicate measurements of each sample in ≥3 independent experiments. ** is for *p* < 0.0005 and *** is for *p* < 0.00005, compared to CHO-K1. (**C**) The accumulation of P-, N-, or C-MWNTs by untransfected CHO-K1 cells and transfected CHO + mSRA1 sub-clones A, B, and C was determined after exposing cells to media containing 100 µg/mL of P-, N-, or C-MWNT for 24 h at 37 °C. After incubation, the cells were washed, cell counts were determined using a Coulter Particle Counter, and MWNTs were extracted and quantified using the SDS-PAGE method. Numbers above the bars are the average fg MWNTs per cell. Data are the average ± SD of ≥3 independent experiments.

Immunofluorescence FCyt showed that these sub-clones of CHO[mSR-AI] cells stably expressed transfected murine surface SR-A1 at various levels, all greater than the background level of the non-transfected wild type CHO-K1 cells (Figure 2A). MFI comparisons indicated that surface SR-A1 levels were increased significantly over wild type CHO-K1 cells, by ~7-fold for CHO + mSRA1.A and CHO + mSRA1.B cells, and by ~3-fold for CHO + mSRA1.C cells (Figure 2B).

To assess the contribution of SR-A1 to MWNT uptake, P-, N-, and C-MWNTs accumulated by wild type CHO-K1 and transfected CHO + mSRA1.A, CHO + mSRA1.B, and CHO + mSRA1.C cells were determined under the same experimental conditions as described in the previous section for macrophages. Data in Figure 2C demonstrate an increased C-MWNT uptake in all three CHO + mSR-A1 sub-clones, specifically, 358 ± 211, 812 ± 221, and 247 ± 159 fg/cell C-MWNT for CHO + mSRA1.A, CHO + mSRA1.B, and CHO + mSRA1.C, respectively, which corresponds to 6-, 14-, and 4-fold increases relative to the 59 ± 17 fg/cell for CHO-K1 cells. The uptake of P- or N-MWNTs, however, remained minimal for all three CHO+mSR-A1 sub-clones, despite the higher SR-A1 expression levels compared to wild type CHO-K1 cells. Thus, the amount of surface SR-A1 correlates with the selective uptake of C-MWNT, but not P- or N-MWNT in CHO clones expressing SR-A1.

Altogether, the low C-MWNT uptake by cells that lack SR-A1 (MS^−/−^ ZK macrophages, Figure 1) and the high uptake of C-MWNT by cells that over-express SR-A1 (CHO + mSRA1 clones, Figure 2) provide strong evidence that SR-A1 underlies the selective uptake of C-MWNTs in macrophages. To determine the potential physiological consequences of high-level accumulation of C-MWNT by macrophages, the cytotoxicity of PF108 alone, P-, N-, and C-MWNTs in RAW 264.7 cells was compared next using three different assays.

### 3.4. The Effect of MWNT Accumulation on Apoptosis, Proliferation, and Colony Formation Efficiency

The first set of experiments assessed the consequences of MWNT exposure on RAW 264.7 cell proliferation using a standardized crystal violet cell proliferation assay [11,24]. Specifically, the ability of cells to proliferate was determined as a function of P-, N-, or C-MWNT concentration in the media, from 25 up to 200 µg/mL, as well as a function of incubation time of 24, 48, or 72 h. Control cells were incubated in regular culture medium that contained no MWNTs or PF108 and their proliferation measured after incubation was set to 100%.

**Figure 3:**
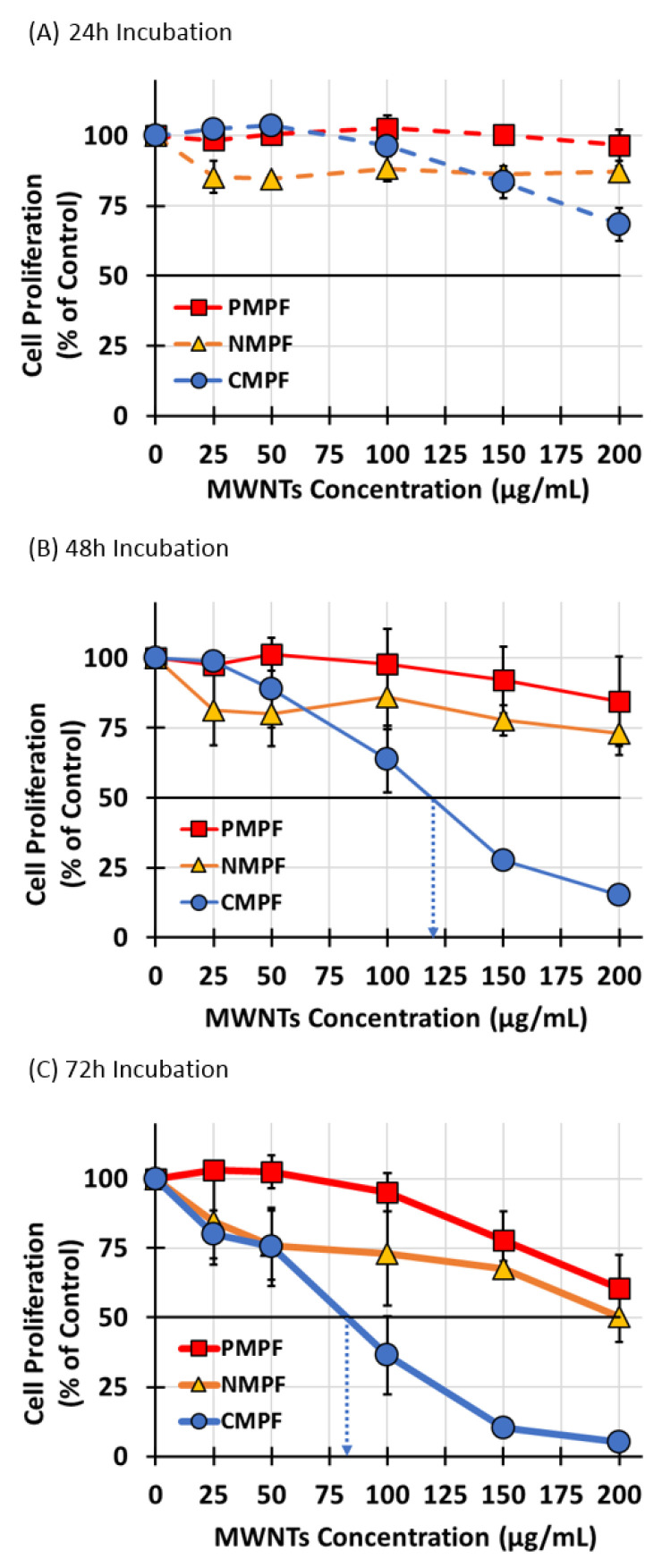
Effects of P-, N-, and C-MWNTs dose and exposure time on RAW 264.7 cell proliferation. RAW 264.7 cells in 48-well plates were incubated in media containing 25, 50, 100, 150, or 200 µg/mL of P-, N-, or C-MWNTs at 37 °C. Cells incubated in regular culture medium in the absence of MWNTs was the untreated control. IC50 values were estimated from a linear regression dose-effect trend line as the concentration of MWNTs needed to inhibit cell proliferation by 50%. Cell proliferation after a (**A**) 24 h, (**B**) 48 h, or (**C**) 72 h exposure to MWNTs was determined by the crystal violet assay where the proliferation of control cells was set to 100%. Dotted arrows indicate estimated IC50 values of 120 and 80 µg/mL for cells treated with C-MWNTs for 48 and 72 h, respectively. Data are the average of quadruplicate measurements per sample in ≥3 independent experiments ± SEM.

After a 24 h incubation (Figure 3A), there was no significant decline in RAW 264.7 cell proliferation for cells with either P- or N-MWNTs at concentrations up to 200 µg/mL, relative to the untreated controls. A slight reduction in proliferation was detected only for cells with C-MWNTs at concentrations of 150 and 200 µg/mL. After a 48 h incubation (Figure 3B), again, no significant effects on cell proliferation were detected for cells with P- or N-MWNTs; however, a greater impact was observed for cells exposed to C-MWNTs, with an estimated IC50 of 120 µg/mL. A longer incubation of 72 h with C-MWNTs aggravated the adverse effect on cell proliferation (Figure 3C), indicated by an IC50 of 80 µg/mL. A 40–50% reduction in cell proliferation for cells with P- or N-MWNTs only became notable after a 72 h exposure and at 200 µg/mL, the highest P- or N-MWNT concentration tested. These data indicate that the IC50 values for exposure to C-MWNTs decrease significantly with time. Therefore, we also studied the effect of MWNTs on RAW 264.7 cells in long-term colony formation assays.

Colony formation assays (CFA), were performed next to assess the impact of various MWNTs on RAW 264.7 cells’ survival upon long-term exposure. This assay tests the ability of single cells to proliferate and form a visible colony under the test condition [25] and has been previously used in studies with CNTs [28,29]. A total of 300 RAW 264.7 cells/well were seeded in a control of regular culture medium or in test media that contained either P-, N-, or C-MWNTs at various MWNT concentrations up to 100 µg/mL, and incubated without disturbance for 8 day to allow colony formation. Colonies were fixed, stained with crystal violet, imaged using a stereomicroscope, and counted.

**Figure 4:**
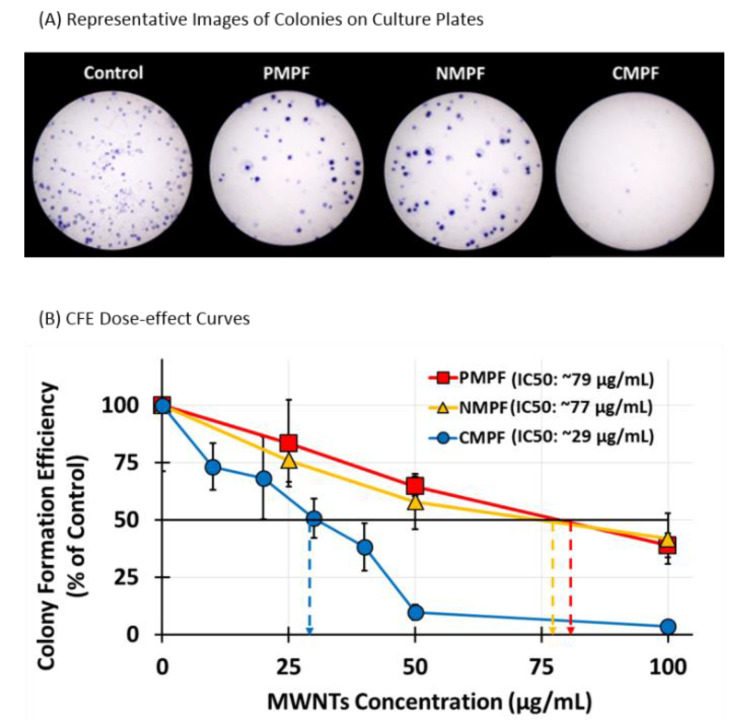
Colony formation efficiency (CFE) of RAW 264.7 cells after 8d of continuous exposure to P-, N-, or C-MWNTs at various concentrations up to 100 µg/mL. 300 RAW 264.7 cells were seeded in 35 mm culture plates with media containing various concentrations of P-, N-, or C-MWNTs up to 100 µg/mL and incubated at 37 °C in a 5% CO_2_ incubator without disturbance for 8 day, then stained as described in Methods. CFE was defined as the ratio of the colony count over the seeded cell count in a plate. The concentration of MWNTs required to inhibit CFE by 50% (IC50) value was estimated from a linear regression dose-effect trend line. (**A**) Representative images of stained colonies in plates with control or cells treated with 100 µg/mL of P-, N-, or C-MWNTs. (**B**) CFE of RAW 264.7 cells incubated in media containing P-, N-, or C-MWNTs for 8 day at 37 °C, relative to untreated control where the CFE was set to 100%. Dotted arrows indicate estimated IC50 values. Data are the average of 6 replicate plates per sample in ≥3 independent experiments ± SD.

Representative images of colonies of the untreated control group and cells incubated with 100 µg/mL P-, N-, or C-MWNTs are shown in Figure 4A. A total of 46 ± 13% of seeded control RAW 264.7 cells formed colonies in 8 day whereas fewer colonies were produced from cells exposed to 100 µg/mL of P- or N-MWNTs. The efficiency of colony formation was near zero for C-MWNT-treated cells. The % colony formation efficiency (CFE) was calculated for each sample, relative to the number of colonies produced by the untreated control set to 100%. A dose-effect curve was plotted for each MWNT type in Figure 4B and demonstrates a dose-dependent decline for CFE. The IC50 values for cells exposed continuously to P-, N-, and C-MWNTs for 8 day were 79, 77, and 29 µg/mL, respectively. The IC50 of 29 µg/mL for the effect of C-MWNT on viability suggests that even lower concentrations could have some adverse effects on cells after long-term exposure.

Apoptosis was also assessed after a 24 h incubation with media containing either 0.1 mM PF108 alone, or 100 µg/mL of P-, N-, or C-MWNTs. Untreated cells provided a negative control and cells treated with 100 nM CPT, a known DNA topoisomerase I inhibitor that causes DNA double-strand breaks [30], were used as positive controls for apoptosis. After the treatments, apoptotic, necrotic, and dead cells were detected using an apoptosis/dead cell detection kit with Annexin V-FITC and PI, according to the manufacturer’s instruction. The % of viable, apoptotic, necrotic, and dead cell fractions in 10,000 cell counts analyzed per sample were acquired using an FCyt cell analyzer. There was negligible necrosis and a background of 4% apoptosis in negative control cells. In CPT treated positive control cells, almost 53% of the cells were apoptotic while necrosis was still low (Figure S1A). Only a low level of apoptosis (11.4–18.0%) was detected in cells exposed to PF108 surfactant alone or to the various MWNTs. The mean % apoptotic cell fractions are plotted as bar graphs in SI Figure S1B to further illustrate that regardless of high accumulation, C-MWNTs had only a mild apoptotic effect on cells in the first 24 h exposure. We next looked at what consequences C-MWNT accumulation via SR-A1 receptors might have on functions related to this receptor.

### 3.5. Treatment of Cells with C-MWNTs, but Not P- or N-MWNTs, Depletes Surface SR-A1

Phagocytosis is important for alveolar macrophages to remove foreign particles such as invading pathogens and dust from the lung. The interaction of C-MWNTs with SR-A1 receptors, and subsequent accumulation in cells, could impair macrophage phagocytic activity. The experiments described in the following sections address: (1) whether the accumulation of C-MWNTs via SR-A1 depletes this important receptor from the cell surface; and (2) whether C-MWNT accumulation impairs the ability of cells to phagocytose ligands, such as polystyrene beads, *E. coli*, and oxidized LDL (oxLDL) that are known to interact with SR-A1 [22,31,32,33,34].

**Figure 5:**
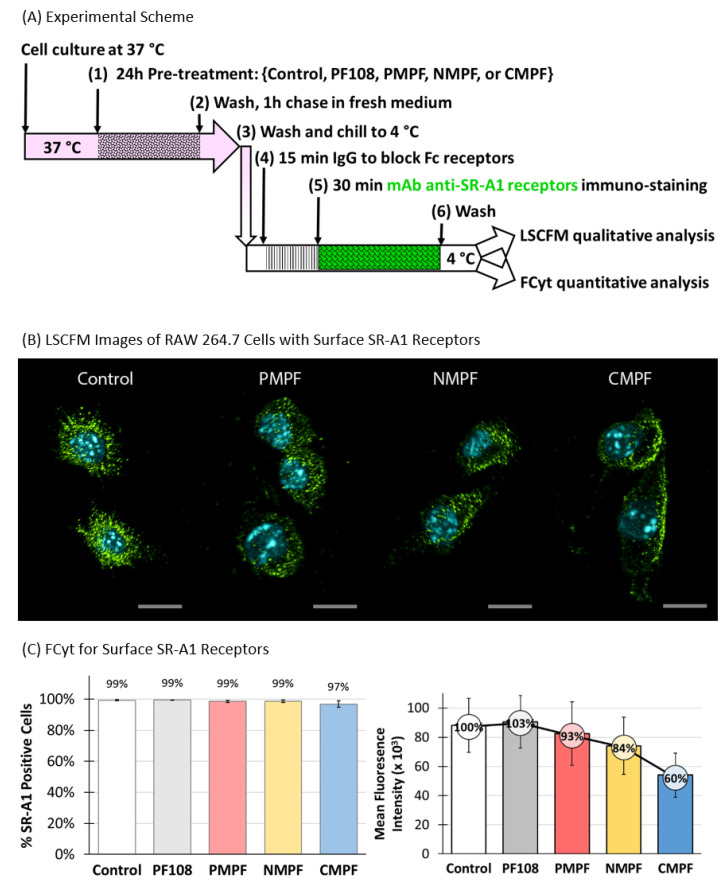
Effects of MWNT accumulation on surface SR-A1 receptor expression in RAW 264.7 cells. RAW 264.7 cells were incubated in media containing 0.1 mM PF108 alone or 100 µg/mL of P-, N-, or CMPF at 37 °C for 24 h. Cells incubated in regular culture medium in the absence of PF108 and MWNTs are the untreated control. Cells were washed and chased in fresh medium at 37 °C for 1 h to promote internalization of MWNTs that may have been on the cell surface. SR-A1 on the cell surface was immunologically detected by either FCyt or LSCFM as described in Methods. Briefly, cells were detached from culture plates with enzyme-free buffer, washed, chilled on ice, and appropriate antibodies were added to cells at 4 °C to prevent internalization of receptors or ligands. (**A**) The timeline scheme outlines the key experimental steps. (**B**) Representative Laser Scanning Confocal Fluorescence Microscopy (LSCFM) images of untreated control and MWNT-treated cells. Blue fluorescence is emitted from Hoechst 33342 stained nucleus and green fluorescence is from Alexa Fluor^®^ 488-conjugated SR-A1 receptors. Scale bars are 10 µm. (**C**) The fraction of positive cells out of a total 20,000 analyzed by flow cytometry was expressed as the % of positive for surface SR-A1 (left panel). The surface SR-A1 receptor expression level is represented as the mean fluorescence intensity of 20,000 cells analyzed per sample (right panel). Percent values inside circles are the mean fluorescence intensity relative to untreated control where the surface SR-A1 receptor level was set to 100%. Data are the mean ± SD of triplicate measurements per sample in ≥3 independent experiments.

Two approaches were used to assess the effects of MWNTs on surface SR-A1 levels in RAW 264.7 cells, a qualitative immunofluorescence microscopy (IFM) assay and a quantitative FCyt assay. The timeline schematic in Figure 5A presents the key steps used to prepare samples for the IFM and FCyt experiments (full details described in the Methods section). Briefly, RAW 264.7 cells were incubated in control medium that contained no PF108 or MWNTs or in test media that contained 0.1 mM PF108 alone or 100 µg/mL of P-, N-, or C-MWNTs at 37 °C for 24 h, washed extensively, chased in fresh medium for 1 h at 37 °C, and then chilled to 4 °C. Half of the cells were incubated with a green Alexa Fluor^®^ 488-conjugated mAb specific for mouse SR-A1 and the other half with an Alexa Fluor^®^ 488-conjugated isotype control mAb as a negative control, at 4 °C for 30 min in the dark. The cells were then prepared either for IFM or FCyt as described in Methods.

Representative LSCFM images of cells stained with Alexa Fluor^®^ 488-conjugated mAbs for SR-A1 and Hoechst dye for the nucleus are shown in Figure 5B. Green fluorescence marking surface SR-A1 was in punctate spots, noted previously and attributed to the localization of SR-A1 in lipid rafts on the cell surface [33]. After C-MWNT treatment, considerably fewer green punctate spots were observed and the spots appeared mainly at cell margins where the cell membrane was attached to the glass coverslip. The untreated control cells appeared to have abundant SR-A1 stains while cells treated with P- and N-MWNTs had slightly less SR-A1 on their surface (Figure 5B). See SI Figure S2 for 360° rotating video clips of representative control and C-MWNT-treated RAW 264.7 cells stained with green fluorescent mAbs against surface SR-A1. None of the cells stained with isotype control mAbs emitted detectable green fluorescence signal, which indicates negligible background fluorescence in this assay (data not shown). These data suggested that SR-A1 might be depleted from the cell surface in RAW 264.7 cells pre-exposed to C-MWNTs, and this was next tested by quantitative FCyt assay.

Cells with a green fluorescence intensity >1 × 10^4^ in the FCyt assay were considered positive for surface SR-A1. Results (Figure 5C, left panel) indicated that ≥97% of RAW 264.7 cells tested positive for surface SR-A1, regardless of any pre-treatment or not. However, as previously noticed in the LSCFM images (Figure 5B), not all SR-A1 positive cells express the same level of surface SR-A1 (Figure 5C right panel). Exposing cells to PF108 alone for 24 h had no effects on their surface SR-A1 expression. A mild reduction in surface SR-A1 expression, of 7% and 16%, was detected in cells pre-treated with either P- or N-MWNTs, respectively, compared to the untreated control cells. A 40% reduction of surface SR-A1 was detected in RAW 264.7 cells pre-treated with 100 µg/mL C-MWNTs for 24 h (Figure 5C, right panel). One interpretation of these data is that notable amounts of surface SR-A1 are depleted from the cell surface upon exposure to C-MWNTs over time. Alternatively, it is possible that cell-associated MWNTs interfere with quantifying surface SR-A1, by quenching the antibody fluorophore signal or by preventing the binding of the reporter antibody in the FCyt experiments. To assess the alternative possibilities, further control experiments were performed, as described next.

### 3.6. MWNTs Do Not Interfere with Immunofluorescence FCyt Assays for Surface SR-A1

To test whether surface-bound MWNTs interfered with the detection of SR-A1 receptors, RAW 264.7 cells were exposed to MWNTs at 4 °C for 1 h, washed to remove unbound MWNTs, and incubated in fresh medium for 1 h at 4 °C. Control cells were kept in medium in the absence of MWNTs at 4 °C. Cells were immuno-stained with fluorescent-conjugated monoclonal anti-SR-A1 antibodies and analyzed for the presence of surface-SR-A1 by FCyt (See SI Figure S3A for a timeline of the treatment). The fluorescent signal in cells treated with MWNTs at 4 °C was compared with the signal from untreated control cells. Interference would be indicated if the signal is attenuated by surface bound MWNTs in the MWNT-treated cells. There was no effect of exposing cells to any type of MWNT at 4 °C on the mean fluorescence intensity of the anti-SR-A1 antibody compared to the untreated control (SI Figure S3B, left side).

Furthermore, to determine whether internalized MWNTs could interfere with the detection of surface anti-SR-A1 immunofluorescence, cells were exposed to MWNTs at 4 °C for 1 h to allow binding, washed to remove unbound material, and then chased at 37 °C for 1 h to permit internalization (see SI Figure S3A). The results in SI Figure S3B, right side, indicate that the internalized MWNTs did not reduce the mean fluorescent signal of the anti-SR-A1 antibody compared to the control. Altogether, these data suggest that neither surface-bound nor internalized MWNTs block the binding of anti-SR-A1 antibody or quench the fluorescent signals of the antibody in the FCyt assay. Thus, the observed 40% reduction of surface SR-A1 in RAW 264.7 cells exposed to C-MWNTs for 24 h is most likely due to a depletion of the SR-A1 receptors from the cell surface over time.

Considering that the accumulation of C-MWNTs by RAW 264.7 cells appears to reduce the expression of SR-A1 on the cell surface, there should be a corresponding effect on the uptake of other SR-A1 receptor ligands. To test this, we next compared the uptake of polystyrene beads, *E. coli*, and oxLDL by untreated control and MWNT-treated RAW 264.7 cells.

### 3.7. Accumulation of C-MWNTs, but Not P- or N-MWNTs, Reduces Uptake of Polystyrene Beads

**Figure 6:**
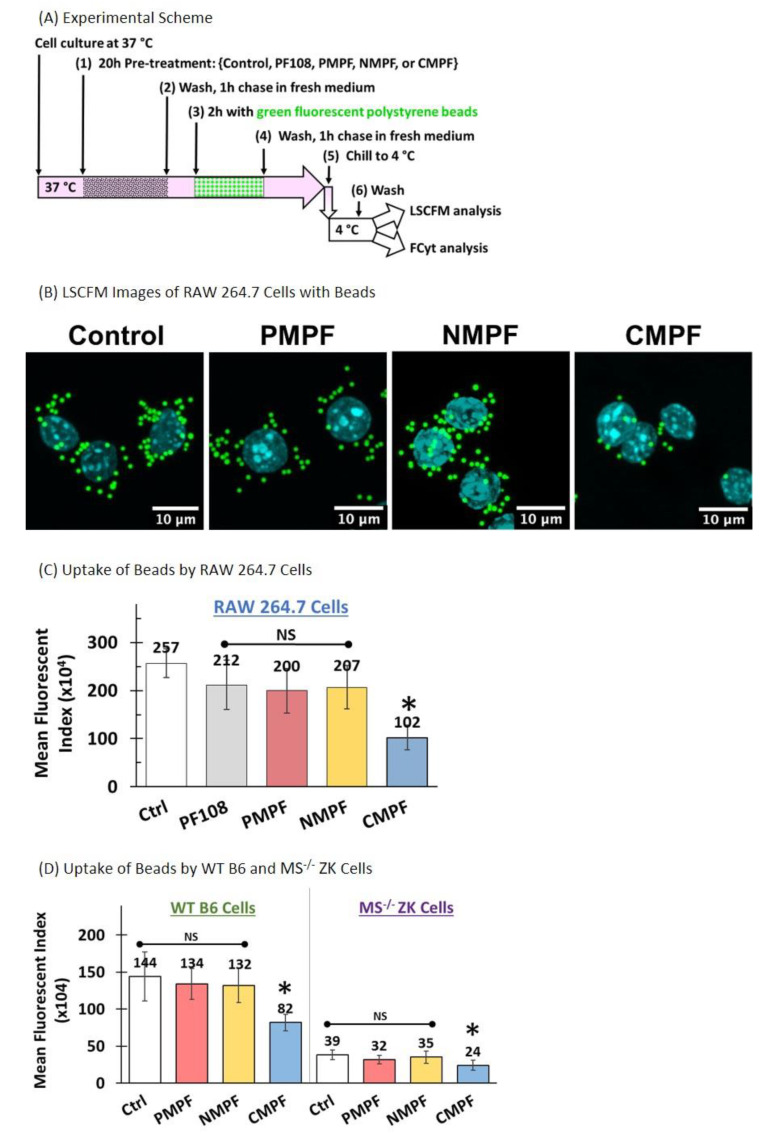
Effects of MWNT accumulation on subsequent phagocytosis of polystyrene beads in murine alveolar macrophage cell lines. RAW 264.7, wild type B6, and MS^−/−^ ZK cells were incubated in media containing 100 µg/mL of P-, N-, or C-MWNTs at 37 °C for 20 h. Untreated control cells were incubated in regular culture medium in the absence of PF108 surfactant or MWNTs. Cells were prepared for LSCFM or FCyt as described in Methods. (**A**) The timeline scheme outlines the key experimental steps. (**B**) Representative LSCFM images of untreated control and MWNT-treated cells. Blue fluorescence is emitted from Hoechst 33342 stained nuclei and green fluorescence is from phagocytosed polystyrene beads. (**C**) Mean fluorescence index of RAW 264.7 control, cells pre-treated with 0.1 mM PF108 alone, or with 100 µg/mL of P-, N-, or C-MWNTs. (**D**) Mean fluorescence index of WT B6 (left panel) and MS^−/−^ ZK (right panel) controls and cells pre-treated with 100 µg/mL of P-, N-, or C-MWNTs. Data are the mean ± SD of duplicate measurements per sample in ≥3 independent experiments. NS is for no significant differences (*p* > 0.05) among test groups, * is for *p* < 0.0005 against control.

Polystyrene beads are negatively charged and are known to bind SR-A1 [22,33,34]. To check the phagocytic ability of RAW 264.7 cells after exposure to different MWNTs, polystyrene beads with internally bound green fluorophores and a nominal diameter of 0.9 μm were used as phagocytic markers. RAW 264.7 cells were pre-incubated with 100 µg/mL P-, N-, or C-MWNTs, or with 0.1 mM PF108 alone at 37 °C for 20 h, washed, and then exposed to 10 µg/mL fluorescent polystyrene beads for 2 h at 37 °C, followed by a 1-h chase at 37 °C in the dark (Figure 6A). The presence of the beads in cells was determined by qualitative assessment using LSCFM and by quantitative FCyt measurement. Representative confocal fluorescence images of untreated control cells and MWNT-treated cells (Figure 6B) visually demonstrate a reduced number of beads in C-MWNT-treated RAW 264.7 cells, whereas cells treated with P- or N-MWNTs have a sparingly reduced number of beads, relative to that of the control cells.

The quantitative results of FCyt analysis (Figure 6C) revealed that the untreated control and cells exposed to either PF108 alone, P-, or N-MWNTs all have high levels of phagocytosed beads, with MFIs of 2.6 × 10^6^, 2.1 × 10^6^, 2.0 × 10^6^, and 2.1 × 10^6^, respectively, indicative of robust phagocytic activity. On the contrary, cells treated with C-MWNTs displayed a significantly reduced level of phagocytosed polystyrene beads, evidenced by a low MFI of 1.0 × 10^6^, ~40% that of the control cells (Figure 6C). These data are consistent with the qualitative observations with LSCFM (Figure 6B and SI Figure S4) and support the idea that the accumulation of C-MWNTs impairs the subsequent uptake of a SR-A1 receptor ligand. Note that in Figure 6B there appears to be fewer, but bright, beads within C-MWNT treated cells, suggesting that quenching is not responsible for the reduced fluorescence signal in cytometry experiments with C-MWNT treated cells.

The possibility that polystyrene beads and PF108-coated C-MWNTs are internalized by the same receptor predicts that they might sometimes both end up within the same phagolysosomes in cells exposed to both ligands. To test this prediction, we used LSCRM to determine whether the distinct Raman signals of polystyrene beads and C-MWNTs co-localized. In LSCRM, a laser scans the cells attached on a coverslip and briefly pauses at each pixel to collect a complete Raman scattering spectrum of the sample, one spectrum per pixel. The scattering signals of C-MWNTs and polystyrene beads in different regions can be extracted, separated, assigned colors, and overlaid to see if they overlap. However, since our results suggest that C-MWNTs and polystyrene beads may use the same receptor, the cells were not simultaneously exposed to both to avoid competition; rather, they were first exposed to C-MWNTs for 2 h, washed, then exposed to beads for 2 h. The cells were then fixed and analyzed by LSCRM as described in the Supplemental Information methods. The Raman signal (red) of cells exposed to beads alone for 2 h mapped to the position of beads seen by bright field microscopy (SI Figure S6, top, left and right). In cells first treated with C-MWNTs for 2 h, washed, then exposed to beads for 2 h, the location of C-MWNTs (green) was visible, as were structures that were yellow, indicating colocalization of beads and C-MWNTs (SI Figure S6, bottom, left and right). The colocalization is partial, as expected because the cells were exposed to the two ligands at separate times. These data provide evidence independent of fluorescence methods that C-MWNTs are internalized and appear with beads in structures that have the expected perinuclear distribution of phagolysosomes.

The effect of MWNTs on the uptake of polystyrene beads was also tested with wild type B6 macrophages, which have SR-A1, and with MS^−/−^ ZK cells, which lack SR-A1. Wild type B6 and MS^−/−^ ZK cells pre-incubated with 100 µg/mL P-, N-, or C-MWNTs at 37 °C for 20 h were washed, chased in fresh medium for 1 h, incubated with 25 µg/mL fluorescent polystyrene beads for 2 h at 37 °C, and washed again prior to FCyt analysis for phagocytosed beads in the cells. Quantitative FCyt results (Figure 6D, left panel) showed that untreated control WT B6 cells and those treated with P- and N-MWNTs have similar phagocytic function and internalized a large number of beads within 2 h of exposure at 37 °C, indicated by their MFI values of 1.4 × 10^6^, 1.3 × 10^6^, and 1.3 × 10^6^, respectively. WT B6 cells treated with C-MWNT, in contrast, displayed a 43% reduction in the uptake of polystyrene beads (MFI 8.2 × 10^5^), compared to the control B6 cells, consistent with the idea that accumulation of C-MWNTs via SR-A1 impairs subsequent uptake of polystyrene beads by WT B6 cells. Unlike the RAW 264.7 or the WT B6 cells, untreated MS^−/−^ ZK cells tested under the same experimental conditions show minimal uptake of polystyrene beads (MFI 3.9 × 10^5^) by the untreated control ZK cells, verifying that SR-A1 are essential for the phagocytosis of polystyrene beads (Figure 6D, right panel). MS^−/−^ ZK cells treated with P-, N-, or C-MWNTs also showed minimal uptake of polystyrene bead (MFIs 3.2 × 10^5^, 3.5 × 10^5^, and 2.4 × 10^5^, respectively).

Replicate experiments of WT B6 and SR-A1-deficient MS^−/−^ ZK cells were analyzed using LSCFM. SI Figure S4 displays representative confocal fluorescence images of B6 and ZK cells with phagocytosed green fluorescent beads and nuclei stained with blue fluorescent Hoechst 33342 dye. The qualitative visual assessment of WT B6 and MS^−/−^ ZK cells using LSCFM (SI Figure S4) confirms the quantitative FCyt results (Figure 6D) for these cells under the same experimental conditions. Altogether, the attenuated uptake of an SR-A1 ligand by cells pre-treated with C-MWNTs correlates well with the observation that C-MWNT accumulation appears to deplete SR-A1 from the cell surface. This correlation was further tested using a bacterial SR-A1 ligand.

### 3.8. Accumulation of C-MWNTs, but Not P- or N-MWNTs, Impairs Subsequent E. coli Uptake

**Figure 7:**
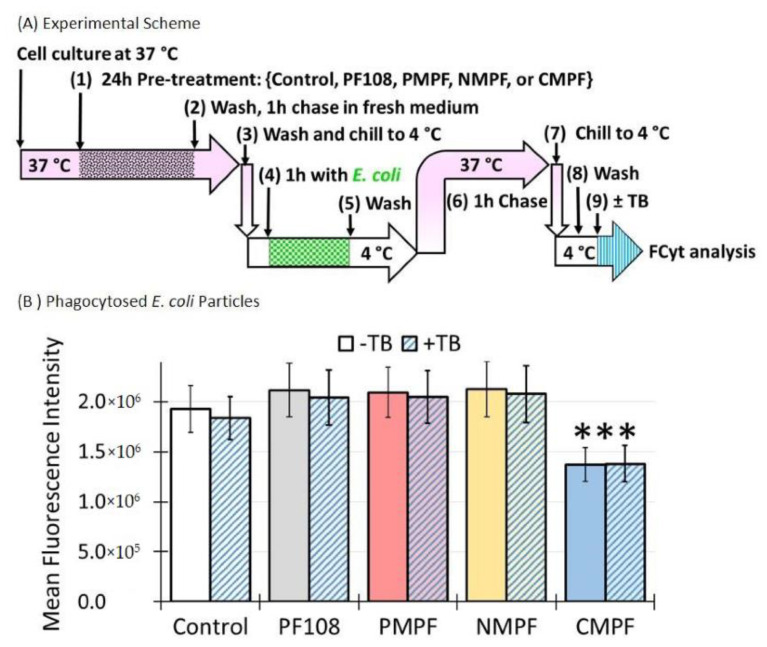
A 24 h exposure of RAW 264.7 cells to C-MWNTs, but not P- or N-MWNTs, impairs subsequent phagocytosis of *E. coli*. RAW 264.7 cells were incubated in media containing 100 µg/mL of P-, N-, or C-MWNTs, or 0.1 mM PF108 alone at 37 °C for 24 h. The cells were chilled to 4 °C and exposed to heat-killed Alexa Fluor^®^ 488-conjugated *E. coli*, at 30 *E. coli* particles per cell, for 1 h at 4 °C, washed, chased for 1 h at 37 °C to allow phagocytosis, washed again, and analyzed by FCyt as described in Methods. Cells with a fluorescence intensity greater than the background auto-fluorescence were considered positive for phagocytosed *E. coli*. Flow cytometric measurements of a sample were followed immediately by consecutive analysis in the presence of 0.1% trypan blue dye, as described in Methods. (**A**) The timeline scheme outlines the key experimental steps. (**B**) *E. coli* phagocytosed by untreated control, PF108-treated, MWNT-treated cells. Mean fluorescence intensity was measured in the absence (−TB) and subsequent presence (+TB) of 0.1% trypan blue. *** is for *p* < 0.0005. Data are the mean ± SD of triplicate measurements per sample in ≥3 independent experiments.

Alveolar macrophages play a key role in host defense against invading pathogens where SR-A1 is critical for bacteria clearance [31,35]. In the following experiments, the potential adverse impacts of C-MWNT accumulation in alveolar macrophage-derived RAW 264.7 cells on *E. coli* clearance were investigated. A schematic of the experimental approach is shown in Figure 7A. Briefly, RAW 264.7 cells were incubated with 100 µg/mL of the indicated MWNTs for 24 h at 37 °C, chased for 1 h at 37 °C and then exposed to heat-killed, green fluorescent-conjugated *E. coli* particles with a multiplicity of infection (MOI) of 30 at 4 °C for 1 h in the dark. Cells were washed to remove unbound *E. coli* particles, placed in fresh medium at 37 °C for 1 h to allow phagocytosis, washed, and analyzed for *E. coli* inside the cells by FCyt. To verify that the fluorescence measured under such conditions was from internalized *E. coli*, cells were treated with TB, which is known to quench extracellular fluorescent signals but has no effect on internalized fluorescence [26,27]. To confirm that TB was an effective quencher of fluorescent *E. coli,* the bacteria were directly exposed to TB in solution and the quenching was assessed by cytometry. The results of this control experiment indicated that the fluorescent signal was quenched by 94%, as shown in SI Figure S5.

The results in Figure 7B revealed that pre-treatment of cells with PF108, P-MWNTs, or N-MWNTs did not reduce their MFI compared to the control cells. In addition, the fluorescence was unaffected by TB, evidence that the fluorescence was from internalized *E. coli* in these cells. However, cells pre-treated with C-MWNTs had a 30% reduction in MFI, even though the fluorescence was, again, highly resistant to quenching by TB. These results support the idea that C-MWNT accumulation impairs *E. coli* clearance by macrophages. In addition, the resistance to TB quenching in the control and cells pre-treated with MWNTs also implies that the plasma membrane of the cells was intact.

### 3.9. Reduced OxLDL Uptake by RAW 264.7 Cells Pre-Exposed to C-MWNTs, but Not P- or N-MWNTs

**Figure 8:**
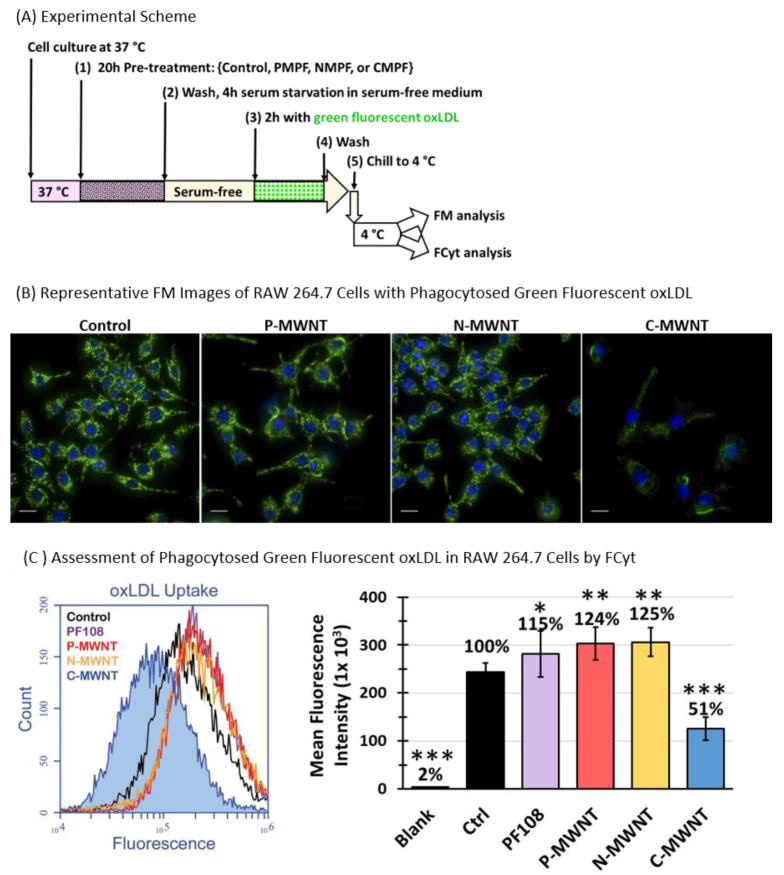
Reduced fluorescent oxLDL uptake by RAW 264.7 cells pre-treated with C-MWNTs, but not P- or N-MWNTs. RAW 264.7 cells were incubated in media containing either 0.1 mM PF108 alone or 100 µg/mL of P-, N-, or C-MWNTs at 37 °C for 24 h. Untreated control cells were incubated in regular culture medium in the absence of PF108 surfactant or MWNTs. After the pre-treatments, cells were washed and serum starved for 4 h in serum-free medium before incubation in fresh serum-free medium containing Alexa Fluor^®^ 488-conjugated oxLDL (1:20 dilution for FM and 1:40 dilution for FCyt) for 2 h at 37 °C. Cells were washed again, chilled to 4 °C, and prepared for FM or FCyt as described in Methods. (**A**) The timeline schematic outlining the key experimental steps. (**B**) Representative epi-fluorescence images of the control and treated cells. Blue fluorescence is emitted from Hoechst 33342 stained nuclei and green fluorescence is from internalized Alexa Fluor^®^ 488-conjugated oxLDL. (**C**) Representative fluorescence histograms of control cells (black), cells pre-treated with 0.1 mM PF108 (purple), 100 µg/mL of P-MWNTs (red), N-MWNTs (yellow), or C-MWNTs (blue) are plotted on the left. The internalized oxLDL is represented as the mean fluorescence intensity of a total of 10,000 cells analyzed per sample and plotted as a bar graph on the right. Data are the mean ± SD of triplicate samples in ≥4 independent experiments. Percent values shown above the bars indicate mean fluorescence intensity relative to untreated control cells where the level of oxLDL was set to 100%. * is for *p* < 5.0 × 10^−2^, ** is for *p* < 5.0 × 10^−5^, and *** is for *p* < 5.0 × 10^−10^ against control.

SR-A1 is one of the three major SRs involved in the recognition and uptake of oxLDL by macrophages, in addition to CD36 and LOX-1 [36]. The uptake and the subsequent intracellular accumulation of oxLDL have been shown to promote the transformation of lipid-laden macrophages to foam cells with proatherogenic effects [36,37,38]. The effect of MWNTs on the uptake of oxLDL was tested with RAW 264.7 cells using the schematic experimental approach shown in Figure 8A. Cells were incubated in media containing either 0.1 mM PF108 alone, 100 µg/mL P-, N-, or C-MWNTs, or in control medium that contained no PF108 or MWNTs for 20 h at 37 °C. After this pre-treatment, the cells were washed and incubated in fresh serum-free medium for 4 h at 37 °C to remove lipid components in the culture medium and then exposed to Alexa Fluor^®^ 488-conjugated oxLDL for 2 h at 37 °C in the dark. Cells were washed and assessed for phagocytosed oxLDL by epi-fluorescence microscopy (FM) and FCyt analysis. Representative fluorescence images of untreated control and MWNT-treated cells are shown in Figure 8B. Active uptake of fluorescent oxLDL was readily visible in untreated control and cells treated with P- or N-MWNTs, whereas green fluorescence was reduced in C-MWNT-treated cells, correlating C-MWNT accumulation with impaired oxLDL uptake by RAW 264.7 cells.

Quantitative results of FCyt analysis (Figure 8C) confirmed the qualitative FM observations shown in Figure 8B. The untreated control and cells exposed to either PF108 alone, P-, or N-MWNTs all had active oxLDL uptake, with mean fluorescence intensities of 2.5 × 10^5^, 2.8 × 10^5^, 3.0 × 10^5^, and 3.1 × 10^5^, respectively, indicative of robust phagocytic activities in these cells. On the contrary, cells with accumulated C-MWNTs displayed a significantly lower fluorescence intensity of 1.3 × 10^5^, indicative of a reduced activity (by ~50%) for oxLDL uptake (Figure 8C). These data, again, support the idea that the accumulation of C-MWNTs impairs the subsequent uptake of SR-A1 receptor ligands.

The concern that C-MWNTs may have quenching effects on the internalized fluorescent oxLDL was addressed with independent oxLDL uptake experiments using non-fluorescent oxLDL. The development of oil droplets from oxLDL uptake by cells was detected visually and measured quantitatively based on an ORO staining assay [39,40,41], with optimization detailed in the Methods section. RAW 264.7 cells seeded on glass coverslips were pre-treated either with regular culture medium as control or with test media containing 100 µg/mL of P-, N-, or C-MWNTs at 37 °C for 24 h. After pre-treatment, cells were washed and serum-starved for 4 h before exposure to non-fluorescent oxLDL for 16 h. The uptake of oxLDL was detected as red oil droplets stained with ORO dye. The red stained oil droplets were readily visible under a bright-field microscope with LED transmitted light and a 40× magnification objective lens. In addition, the ORO stain was eluted in isopropanol and measured quantitatively as absorbance intensity at 510 nm.

Representative ORO stained images of control and MWNT-treated cells, with and without oxLDL exposure, are shown in SI Figure S7B. Without oxLDL exposure, no ORO stains were visible in either control or any of the MWNT-treated cells, indicating that endogenous oil droplets were successfully depleted during the serum starvation period. On the contrary, robust oxLDL uptake was evidenced by the red ORO stain in cells allowed to take up oxLDL present in the media. Notice that internalized MWNTs were clearly visible as dark gray vesicular structures inside cells pre-treated with C-MWNTs that may obstruct visual assessment of red oil droplets in these cells. Thus, cell-associated ORO was determined by extracting the dye from cells with isopropanol, and the eluent was then transferred to new plates and measured spectrophotometrically free of potential MWNT interference. Results of the quantitative oxLDL uptake assessment, represented by the relative ORO stain intensities of the untreated control and cells pre-treated with MWNTs, indicated that oxLDL uptake was reduced by ~30% in cells pre-treated with C-MWNTs, whereas P- or N-MWNTs had no effect on subsequent oxLDL uptake (SI Figure S7C). These results agree with data in Figure 8 using fluorescent oxLDL and validate the finding that accumulation of C-MWNTs in RAW 264.7 cells affects subsequent phagocytosis of oxLDL.

## 4. Discussion

Our present hypothesis for the selective interaction of PF108-coated C-MWNTs with macrophages is that the primary determinant of the interaction is oxidative functionalities on nanotubes, which could include carboxylation, hydroxylation, and other carbon-oxygen bonded groups. This idea is supported by our previous work comparing various physical properties of the carbon nanotubes that are highly accumulated by cells. Both carboxylated single-walled carbon nanotubes (C-SWNTs) as well as C-MWNTs are highly accumulated, but not their pristine counterparts, suggesting that neither nanotube diameter nor chirality are critical parameters for accumulation [11]. Moreover, both short and long C-MWNTs were accumulated, so length is apparently not a factor [11]. It is unlikely that the non-ionic Pluronic^®^ coat on the nanotubes influences interactions with cells because both carboxylated and pristine nanotubes bear the same coat yet they bind and accumulate differently. It is also notable that a protein corona is not required for receptor interaction as binding to cells occurs at 4 ℃ in the absence of serum [11]. The zeta potentials of the carboxylated nanotubes are more negative than their pristine counterparts, as expected, and a negative charge is a common physical feature related to carboxylation shared by nanotubes that are highly accumulated. Members of Class A SRs interact with certain anionic ligands and polyanions such as dextran sulfate often antagonize this interaction. Dextran sulfate and similar compounds were previously noted to affect the response of cells to carbon nanotubes, suggesting that scavenger receptors may be involved [42,43,44]. In addition, Singh et al. observed that increased carboxylation of MWNTs correlated with MWNT accumulation at 37 °C by RAW 264.7 cells, and accumulation was inhibited by dextran sulfate [45]. We previously noted that dextran sulfate partially inhibits the binding of C-MWNTs to cells at 4 °C and accumulation at 37 °C, suggesting that SR-A members might be C-MWNT receptors [11]. However, data from inhibitor studies can be difficult to interpret when assigning a ligand interaction to a specific receptor, especially with scavenger receptors that often share similar binding domain structures. One main objective of the present study was to test the hypothesis that SR-A1 is a receptor for C-MWNTs using cell lines that either do or do not express SR-A1, a more informative approach than using inhibitors of ligand binding.

ZK alveolar macrophages, derived from SR-A1/MARCO deficient mice [22], were verified to lack SR-A1 and MARCO by FCyt. B6 cells, a wild type alveolar macrophage control, were positive for SR-A1, as was the established alveolar macrophage-derived cell line RAW 264.7. Both B6 and RAW 264.7 cells robustly accumulated C-MWNTs during a 24 h exposure, while ZK cells did not, thus correlating the presence of SR-A1 with the capacity to accumulate C-MWNTs. None of the cells expressed significant levels of MARCO making it unlikely that MARCO contributes to C-MWNT uptake by B6 and RAW 264.7 cells. However, this does not imply that MARCO cannot bind C-MWNTs because MARCO, like SR-A1, contains a collagenous domain believed to bind polyanionic ligands. In fact, it was observed that ectopic expression of MARCO in CHO cells enhanced the uptake of MWNTs [46]. Therefore, MARCO may be involved in C-MWNTs uptake by other cell types that express this scavenger receptor. N-MWNTs, like P-MWNTs, did not accumulate in any of the cells tested, consistent with the idea that the anionic carboxyl groups of C-MWNTs are a determinant of receptor-mediated uptake.

CHO[mSR-AI] cells that express SR-A1 were studied to determine whether SR-A1 expression caused a gain in the capacity to accumulate C-MWNTs. As received CHO[mSR-AI] cells were re-cloned and all three clones were positive for surface SR-A1 by FCyt compared to wild type CHO-K1 cells and all three clones accumulated C-MWNTs, but not P-MWNTs or N-MWNTs. In summary, cells that did not express SR-A1 did not accumulate C-MWNTs, while those that expressed SR-A1 did accumulate C-MWNTs, strong evidence that SR-A1 is a C-MWNT receptor and functions in the receptor-mediated uptake of C-MWNTs.

A corona of proteins derived from biological fluids is often believed to be a major determinant in the interaction of engineered nanoparticles with cells [47,48,49,50]. For C-MWNTs coated with Pluronic^®^, since binding and uptake can occur in the absence of serum, the major determinant of C-MWNT binding and accumulation appears to be the oxidized functionalities and not a protein corona on the MWNT surface; nevertheless, serum is a dose dependent inhibitor of binding [11]. This suggests that something in serum either interacts with C-MWNTs, with SR-A1, or both, to influence accumulation. Thus, although a protein corona derived from serum is not necessary for C-MWNTs to bind to cells, it may still affect interactions if present. Moreover, it should be possible to increase the complexity of the model system by adding a defined protein corona to C-MWNTs to determine whether carboxylation still contributes to the interaction of nanotubes with SR-A1 and whether the protein expands interactions to other receptors.

A second objective of this study was to assess the effects on cell viability of short- and long-term exposure of SR-A1-positive cells to MWNTs. Apoptosis was measured by flow cytometry using an apoptotic/dead cell kit after a 24 h exposure of RAW 264.7 cells to MWNTs (100 µg/mL), and there was no significant difference among cells treated with PF108 alone and cells treated with C-MWNTs, P-MWNTs and N-MWNTs, compared to a positive control of CPT treated cells where apoptosis was obvious. In previous work, we found that a 24 h exposure to C-MWNTs had little effect on cell proliferation [11], and here we compared cell proliferation as a function of MWNT concentration at 24, 48, and 72 h. Beyond 24 h, C-MWNTs more adversely affected proliferation than P-MWNTs and N-MWNTs. By 72 h, the IC50 for C-MWNTs was ~80 µg/mL whereas P-MWNTs had not reached an IC50 at 200 µg/mL and N-MWNTs had barely reached IC50 at 200 µg/mL, the highest MWNT concentration tested. Because exposure time was an important parameter in toxicity experiments, colony formation efficiency assays were done where cells were continuously exposed to different MWNT concentrations for 8 day. After an 8-d exposure to 100 µg/mL C-MWNTs, there were essentially no colonies formed whereas P-MWNTs and N-MWNTs had ~40% colony formation efficiency compared to the untreated control under the same condition. The IC50 for C-MWNTs was ~29 µg/mL, but there was a reduction in colony formation efficiency even in the range of a few µg/mL, which is important as it suggests that even low doses of C-MWNTs over time may accumulate and adversely affect cells. These data emphasize that C-MWNTs are more toxic than P-MWNTs or N-MWNTs, especially after longer exposures, which is not surprising considering that RAW 264.7 cells accumulate about 80–100 times more C-MWNTs than P-MWNTs or N-MWNTs in 24 h. However, it is also notable that for a given IC-50, the amount of C-MWNTs in the cell on a per cell basis is much higher than for P-MWNTs or N-MWNTs, indicating that on an MWNT weight basis per cell, C-MWNTs appear to be less toxic than P-MWNTs or N-MWNTs. Thus, long-term exposure to MWNTs at lower concentration, regardless of carboxylation, may affect cell viability.

A third objective of this study was to assess what might be the spectrum of physiological consequences to macrophage functions after exposure to MWNTs, specifically related to the interaction of C-MWNTs with SR-A1. One possibility is that internalization of surface SR-A1/C-MWNT complexes could deplete the plasma membrane of SR-A1 receptors. To assess this, cells were treated with MWNTs at 37 °C followed by immunofluorescence detection of surface SR-A1 with a specific antibody, using both qualitative IFM images and quantitative FCyt analysis. In untreated cells and those treated with P-MWNTs or N-MWNTs, SR-A1 was present in punctate spots that are believed to be receptors clustered in lipid rafts scattered over the cell surface [33]. However, after C-MWNT treatment, the overall surface SR-A1 signal was reduced and redistributed mainly to the cell margins. The reason for this abnormal distribution is not clear, but it might adversely affect SR-A1 function. In addition, the fluorescent signal from C-MWNT-treated cells by FCyt analysis was reduced by about 40% whereas the signal from P-MWNT- or N-MWNT-treated cells was much less affected. A concern in the interpretation of the FCyt results is that C-MWNTs might interfere with the fluorescence signal, even though the internalized C-MWNTs are mainly within intracellular vesicles and the fluorescent anti-SR-A1 is on the cell surface. To address this concern, cells were treated with MWNTs at 4 °C to allow binding, then chased for an hour at either 37 or 4 °C. The 37 °C chase was to allow surface MWNTs to be internalized and the 4 °C treatment was to keep them on the cell surface. Fluorescent monoclonal anti-SR-A1 was then added at 4 ℃ and the fluorescence signal was measured by FCyt. There was no attenuation of the surface fluorescence signal by C-MWNTs, suggesting that C-MWNTs have little influence on the signal of anti-SR-A1 antibody regardless of whether the MWNTs were internalized or on the cells surface. These data are consistent with the idea that internalization of C-MWNTs via SR-A1 depletes the cell surface of this important receptor.

The ability of C-MWNT-treated RAW 264.7 cells to respond to several known SR-A1 ligands was also examined. The internalization of fluorescent polystyrene beads measured by FCyt was impaired by over 50% after C-MWNT treatment, but not markedly affected after P-MWNT or N-MWNT treatment. Note also that ZK cells lacking SR-A1 failed to accumulate beads when untreated with C-MWNTs, verifying that SR-A1 was a receptor for the beads. We previously reported that the uptake of non-fluorescent polystyrene beads by RAW 264.7 cells treated with C-MWNTs for 2 or 20 h was reduced by 2-fold and 6-fold, respectively, when assayed by directly counting the number of beads in cells [11]. This adds confidence that the decline in the fluorescence signal from internalized beads in C-MWNT-treated cells is not an artifact of fluorescence quenching by cell-associated C-MWNTs.

The uptake of heat-killed Alexa Fluor^®^ 488-conjugated *E. coli* was measured as another model system to assess SR-A1 function after MWNT treatment. RAW 264.7 cells treated with or without MWNTs for 24 h were chilled and incubated with *E. coli* at 4 °C low temperature to load receptors, followed by incubation at 37 ℃ to permit internalization. Cells treated with P-MWNTs or N-MWNTs showed no decline in fluorescence signal whereas cells treated with C-MWNTs showed a 30% decline, evidence that the clearance of a physiologically relevant SR-A1 ligand is affected. In these experiments, the ability of trypan blue to quench Alexa Fluor^®^ 488 was used to verify that the fluorescent *E. coli* were inside the cell sheltered from contact with trypan blue, which does not penetrate the plasma membrane of intact cells.

The final SR-A1 ligand studied was oxLDL. One assay measured the accumulation of fluorescent oxLDL by FCyt and another used a non-fluorescence-based approach to measure the development of fat droplets induced by oxLDL accumulation. Both assays revealed reduced oxLDL accumulation by cells treated with C-MWNTs but not with P-MWNTs or N-MWNTs, confirming that another important function of SR-A1, the clearance of oxLDL, was impaired.

In summary, the uptake of three different SR-A1 ligands was impaired in RAW 264.7 cells by C-MWNT accumulation. One major factor affecting the uptake is likely to be the reduced number of SR-A1 receptors on the cell surface. Impaired functionality related to one or more of these ligands could contribute to the observed reduced viability of C-MWNT-treated cells. Thus, there are likely related adverse consequences affecting other biological functions, in addition to impaired SR-A1 functions demonstrated here, resulting from selective C-MWNT uptake in macrophages.

## 5. Conclusions

The binding and accumulation of Pluronic^®^-coated MWNTs described here provides a model system where key experimental parameters of the interaction of carbon nanomaterials with cells can be controlled. One feature of the approach is the SDS-PAGE assay that quantitates nanotubes extracted from cells, enabling the direct measurement of nanotube binding and accumulation by cells. In addition, binding of MWNTs to cell surface receptors can be determined at 4 ℃ and in the absence of serum where other physiological activities, such as endocytosis, vesicle recycling, enzymatic degradation, or interaction influenced by serum proteins corona were curtailed. The approach permitted the direct observation that carboxylated nanotubes, but not pristine or amino-functionalized counterparts, could selectively bind to and be accumulated by cells, suggesting that there were receptors that recognized carboxylated nanotubes. The results of studies with cells that either over or under express SR-A1 strongly implicate SR-A1 as the key receptor for carboxylated nanotubes. Two lines of evidence also suggest that there are adverse consequences of nanotube accumulation related to SR-A1 functions. First, the uptake of three different SR-A1 ligands (polystyrene beads, *E. coli*, and oxLDL) was impaired in RAW 264.7 cells laden with C-MWNT, emphasizing that pathogen clearance and lipid homeostasis could be affected in macrophages exposed to C-MWNTs. Second, C-MWNT uptake via SR-A1 leads to a concentration and time dependent reduction in the viability of alveolar macrophages. The results of this study may have implications for other oxidized carbon nanomaterials and it would be interesting to know if graphene oxide and carbon-based quantum dots also bind SR-A1.

## Figures and Tables

**Table 1:** Properties of pristine- (P-), amino-functionalized- (N-), and carboxylated-multi-walled carbon nanotubes (C-MWNT) powders and prepared Pluronic^®^ F108-coated MWNT dispersions.

MWNT Product Specification Provided by NanoCyl								MWNT Particles in Pluronic^®^ F-108 Dispersions				
MWNT Product	Batch No.	Carbon Purity (wt.%)	Surface Modification	NH_2_ COOH (wt.%)	Metal Oxide (wt.%)	Average Length (µm)	Average Diameter (nm)	MWNT-PF108 Dispersion	Particle Size		Zeta Potential (mV)	
									HDD (nm)	PDI	Water 25 °C	Medium +10% FBS 37 °C
NC3150™ Pristine (**P-MWNT**)	100426	>95	-	-	<5.0	<1.0	9.5	**PMPF**	114 ± 1.0	0.22	−22.2 ± 2.6	−1.2 ± 0.3
NC31520™ Amino-functionalized (**N-MWNT**)	MEL 160125	>95	-NH_2_	<0.6	<5.0	<1.0	9.5	**NMPF**	108 ± 0.2	0.22	−20.9 ± 0.7	−1.2 ± 0.3
NC31510™ Carboxyl-functionalized (**C-MWNT**)	120828	>95	-COOH	<8.0	<5.0	<1.0	9.5	**CMPF**	86 ± 1.0	0.23	−26.8 ± 1.0	−4.8 ± 0.6

## Supplementary Materials

The following are available online at https://www.mdpi.com/2079-4991/10/12/2417/s1, Figure S1: MWNTs induce mild apoptosis in RAW 264.7 cells. Figure S2: Representative 3D video clips of control and C-MWNT-treated RAW 264.7 cells immuno-fluorescent stained for surface SR-A1 receptors. Figure S3: Surface-bound or phagocytosed MWNTs do not interfere with immunofluorescence FCyt assays for surface SR-A1 receptors on RAW 264.7 cells. Figure S4: Effects of MWNT accumulation on subsequent phagocytosis of polystyrene beads in WT B6 and MS^−/−^ ZK cells assessed by LSCFM. Figure S5: Fluorescence quenching of fluorescent-conjugated, heat killed *E. coli* particles by trypan blue dye. Figure S6: Laser scanning confocal Raman microscopy analysis of RAW 264.7 cells with phagocytosed polystyrene beads in the present or absence of C-MWNTs. Figure S7: Reduced oxLDL uptake by RAW 264.7 cells pre-treated with C-MWNTs, but not P- or N-MWNTs, assessed using ORO staining assays. (Directions to access the SI will be updated in the final version of the manuscript.)
