# Supplementary material for: Selective Uptake of Carboxylated Multi-Walled Carbon Nanotubes by Class A Type 1 Scavenger Receptors and Impaired Phagocytosis in Alveolar Macrophages
Source: Nanomaterials (Basel). 2020 Dec 3;10(12):2417. doi: 10.3390/nano10122417 (PMC7761752; doi:10.3390/nano10122417)
Supplement: Supplementary file 1 [file nanomaterials-10-02417-s001.zip › nanomaterials-988389-update SI 2/SI-nanomaterials-988389.pdf]

Selective Uptake of Carboxylated Multi-Walled Carbon Nanotubes by Class A Type 1 Scavenger Receptors and Impaired Phagocytosis in Alveolar Macrophages

Ruhung Wang [1,2], Rishabh Lohray [1,†], Erik Chow [3,‡], Pratima Gangupantula [1], Loren Smith [2] and Rockford Draper [1,2,*]

1 Department of Biological Sciences, The University of Texas at Dallas, 800 West Campbell Road, Richardson, TX 75080, USA; ruhung.wang@utdallas.edu (R.W.); Rishabh.Lohray@bcm.edu (R.L.); Pratima.Gangupantula@utdallas.edu (P.G.)
2 Department of Chemistry & Biochemistry, The University of Texas at Dallas, 800 West Campbell Road, Richardson, TX 75080, USA; Loren@utdallas.edu
3 Department of Bioengineering, The University of Texas at Dallas, 800 West Campbell Road, Richardson, TX 75080, USA; ec829@cornell.edu
* Correspondence: draper@utdallas.edu
† Present Affiliation: Baylor College of Medicine, School of Medicine, Houston, TX 77030, USA.
‡ Present Affiliation: Cornell University, Meinig School of Biomedical Engineering, Ithaca, NY 14850, USA.

SUPPLEMENTAL MATERIAL

(For Wang et al., Selective Uptake of Carboxylated Multi-walled Carbon Nanotubes by Class A Type 1 Scavenger Receptors and Impaired Phagocytosis in Alveolar Macrophages)

Supplemental Methods

Laser scanning confocal Raman microscopy (LSCRM) of RAW 264.7 cells with and without phagocytosed C-MWNTs and polystyrene beads

4×10^4 RAW 264.7 cells/well seeded in 4-well plates on glass coverslips were incubated at 37 °C under standard cell culture conditions for 24 h prior to experiments. Cells were incubated in control medium without PF108 or MWNTs or in test medium containing PF108-coated C-MWNTs at 100 μg/mL for 2 h at 37 °C. After the pre-treatment, cells were washed thoroughly followed by a 30-min chase in fresh medium at 37 °C. Cells were washed again prior to exposure to non-fluorescence labeled and non-functionalized 0.9 μm polystyrene beads at 25 μg/mL for 2 h

at 37 °C. After the exposure, cells were washed thoroughly again to remove extracellular polystyrene beads in the medium, fixed with 4% (w/v) PFA at room temperature for 15 min, rinsed briefly with water, then allowed to air-dry with cell-side up on glass coverslips at room temperature. Raman images of the cells were acquired with a WITec 500R laser confocal scanning microscopy system equipped with a 532 nm laser and 100 × magnification objective lens (NA = 0.9), similar to methods previously published by our group [1-4]. A bright-field image of the cells on coverslip was acquired to define the x- and y-coordinate system. Cells were scanned using a laser power of 1 mW/cm^2 and an integration time of 0.1 s per spectrum where 22,500 Raman spectra were acquired over a 50 × 50 μm area. After scanning, each Raman spectrum was analyzed independently for the presence of two distinct Raman signals, one specific for polystyrene and one for MWNT with two distinct filters. Specifically, a wavenumber filter (950-1050 cm^{-1}) corresponding to the breathing mode of the phenyl ring in polystyrene beads was used to detect the beads. The summation of CCD counts within the filter region was calculated for each spectrum. A false color was then assigned to the pixel according to the CCD counts and a defined color scale where the pixel with a highest CCD counts was colored in red and the pixel with a lowest CCD counts was colored in black. Similarly, all Raman spectra were also analyzed with a second wavenumber filter corresponding to the D-band signal region of MWNTs (1300-1400 cm^{-1}). The pixel with the highest summation of CCD counts within this D-band filter region was given a false color in green and the pixel with a lowest CCD counts was colored in black. All Raman data analysis, including background subtraction, peak area intensity integration, and images transformation and overlay were performed using *WITec Project 4 Plus* software.

REFERENCES

1. Marches, R.; Mikoryak, C.; Wang, R.H.; Pantano, P.; Draper, R.K.; Vitetta, E.S. The importance of cellular internalization of antibody-targeted carbon nanotubes in the photothermal ablation of breast cancer cells. Nanotechnology 2011, 22, 095101, doi:10.1088/0957-4484/22/9/095101.

2. Wang, R.; Mikoryak, C.; Li, S.; Bushdiecker 2nd, D.; Musselman, I.H.; Pantano, P.; Draper, R.K. Cytotoxicity screening of single-walled carbon nanotubes: detection and removal of cytotoxic contaminants from carboxylated carbon nanotubes. Mol Pharm 2011, 8, 1351-1361, doi:10.1021/mp2001439.

3. Pantano, P.; Draper, R.K.; Mikoryak, C.; Wang, R. Electrophoretic methods to quantify carbon nanotubes in biological cells. In Handbook of Carbon Nanomaterials, D'Souza, F., Kadish, K.M., Eds. World Scientific Publishers: Singapore, 2012; Vol. 3, pp. 84-107.

4. Murali, V.S.; Wang, R.; Mikoryak, C.A.; Pantano, P.; Draper, R.K. The impact of subcellular location on the near infrared-mediated thermal ablation of cells by targeted carbon nanotubes. Nanotechnology 2016, 27, 425102 (425115 pp), doi:10.1088/0957-4484/27/42/425102.

I Figure S1.

(A) Representative Dot Plots of RAW 264.7 Cells in Apoptosis Assay

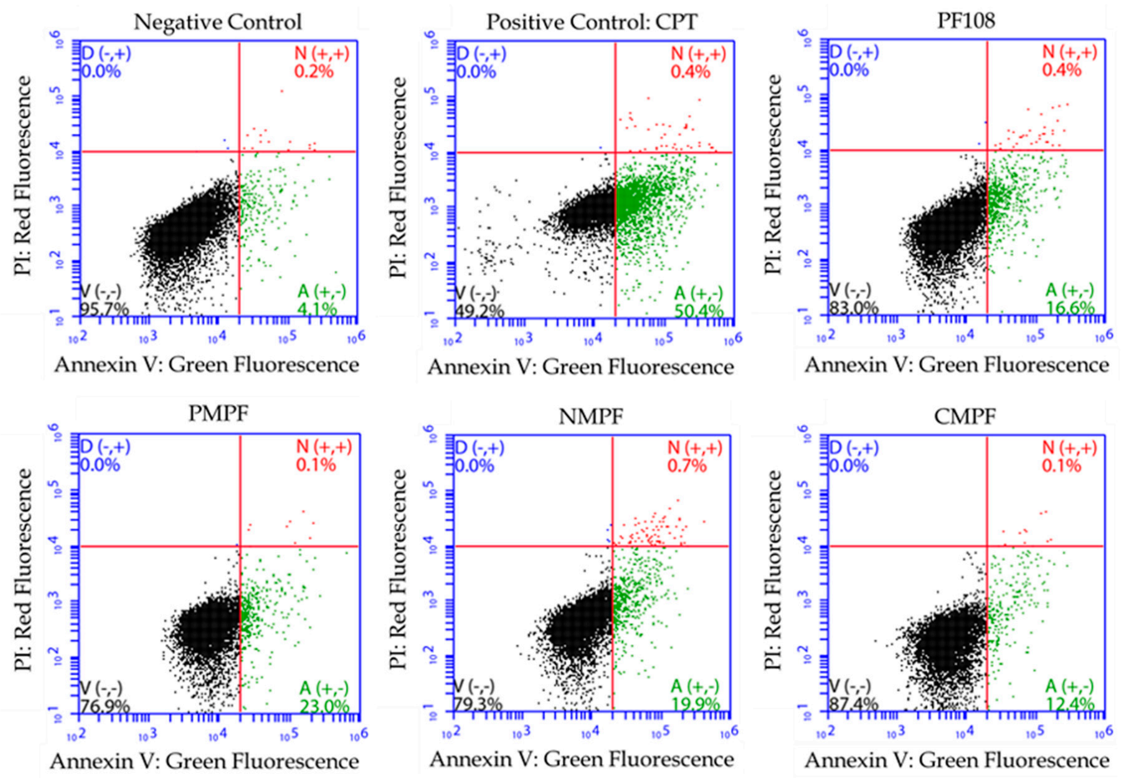

(B) Mean % Apoptotic Cell Population

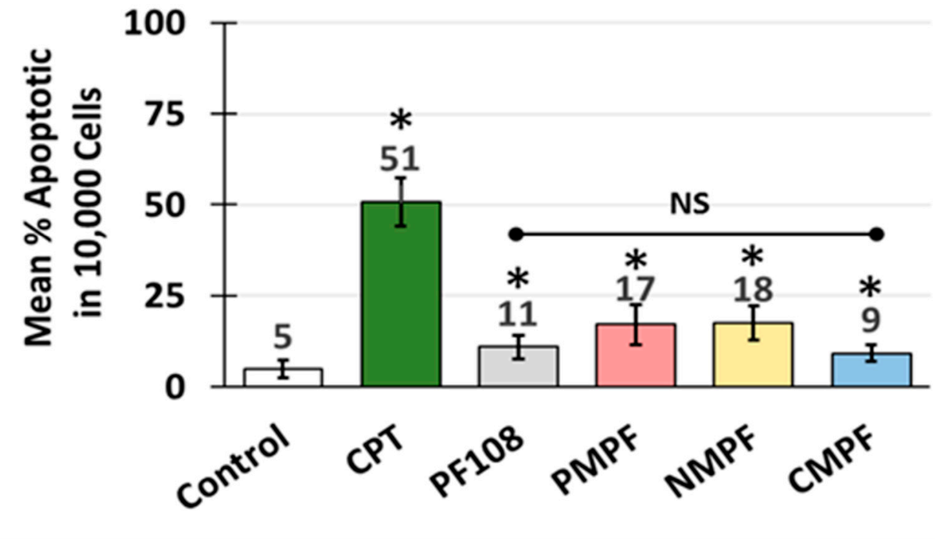

SI Figure S1. MWNTs induce mild apoptosis in RAW 264.7 cells. RAW 264.7 cells were incubated in media containing either 0.1 mM PF108 alone or 100 μg/mL of P-, N-, or C-MWNTs for 24 h at 37 °C. Cells incubated in regular culture medium in the absence of test additives is the untreated negative control. Cells treated with 100 nM camptothecin (CPT) in culture medium were used as positive control for apoptosis. Cells were washed and detached from culture vessel using an enzyme-free buffer. Cells in suspension were assessed for apoptosis/necrosis using an apoptosis detection kit with Annexin V-FITC and propidium iodide (PI) (Invitrogen Cat. # V14242) for flow cytometry analysis. After treatment with annexin V and PI, cells with higher green fluorescence are apoptotic, cells with higher green and higher red fluorescent signals are necrotic, dead cells show higher red but not green fluorescence, and viable cells show little or no fluorescence higher than background auto-fluorescence. The fractions of viable, apoptotic, necrotic, and dead cells in 10,000 counts analyzed per measurement were acquired. (A) Representative dot plots with annexin V-FITC green fluorescence on the x-axis and PI red fluorescence on the y-axis; (B) mean % apoptotic cells in 10,000 analyzed of untreated RAW 264.7 control and cells treated with 100 nm CPT alone, 0.1 mM PF108 alone, 100 μg/mL of P-, N-, or C-MWNT for 24 h at 37 °C. Data is the average of duplicate measurements per sample in ≥3 independent experiments ± SD. * is for $P<0.01$ against negative control and NS is for no significant difference ($P> 0.01$) against cells treated with PF108 alone.

SI Figure S2.

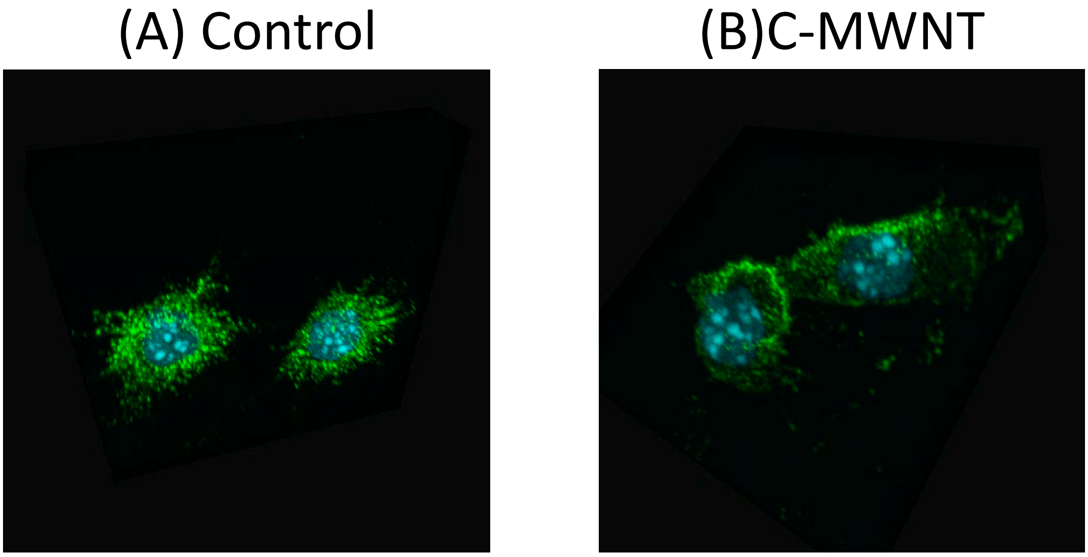

SI Figure S2. Representative 3D video clips of control and C-MWNT-treated RAW 264.7 cells immuno-fluorescent stained for surface SR-A1 receptors. See Figure 5A for a time-line scheme that outlines the key experimental steps in the protocol. RAW 264.7 cells grown on glass cover slips were incubated in medium containing 100 μg/mL of C-MWNT for 24 h at 37°C. Cells incubated in regular culture medium in the absence of MWNTs is the untreated negative control. Cells were washed and chased in fresh medium at 37 °C for 1 h, washed, and chilled on ice. Surface Fc receptors were blocked with mouse IgG. Surface SR-A1 receptors were detected by incubating the cells with Alexa Fluor® 488-conjugated monoclonal rat anti-mouse mSR-A1/MSR antibody specific for SR-A1 receptor at 4 °C for 30 min in the dark. Cells were washed to remove unbound antibodies, incubated with Hoechst 33342 dye, fixed with 4% PFA, and mounted on glass slides. Automatic Z-sectioned fluorescence images of the cells were acquired using a laser scanning confocal fluorescence microscope with 100× objective lens magnification. Blue fluorescence is emitted from Hoechst 33342 stained nuclei and green fluorescence is from Alexa

Fluor® 488 stained SR-A1 receptors. 3D rendering of cells were reconstructed from ~30 confocal images along the Z-axis and 360° rotating images were recorded as video clips using ImageJ software. Representative 360° rotating 3D video clips of RAW 264.7 (A) untreated control cells, and (B) cells treated with 100 µg/mL C-MWNTs for 24 h at 37 °C, immuno-stained with green fluorescent-conjugated mAbs specific for SR-A1 receptors and blue fluorescent Hoechst dye for the nuclei. The untreated control cells have more green surface SR-A1 stain and C-MWNT treated cells have less SR-A1 receptors on their surface. (First click near the images to activate the embedded .pptx file, then double click on the image to activate the video.)

SI Figure S3.

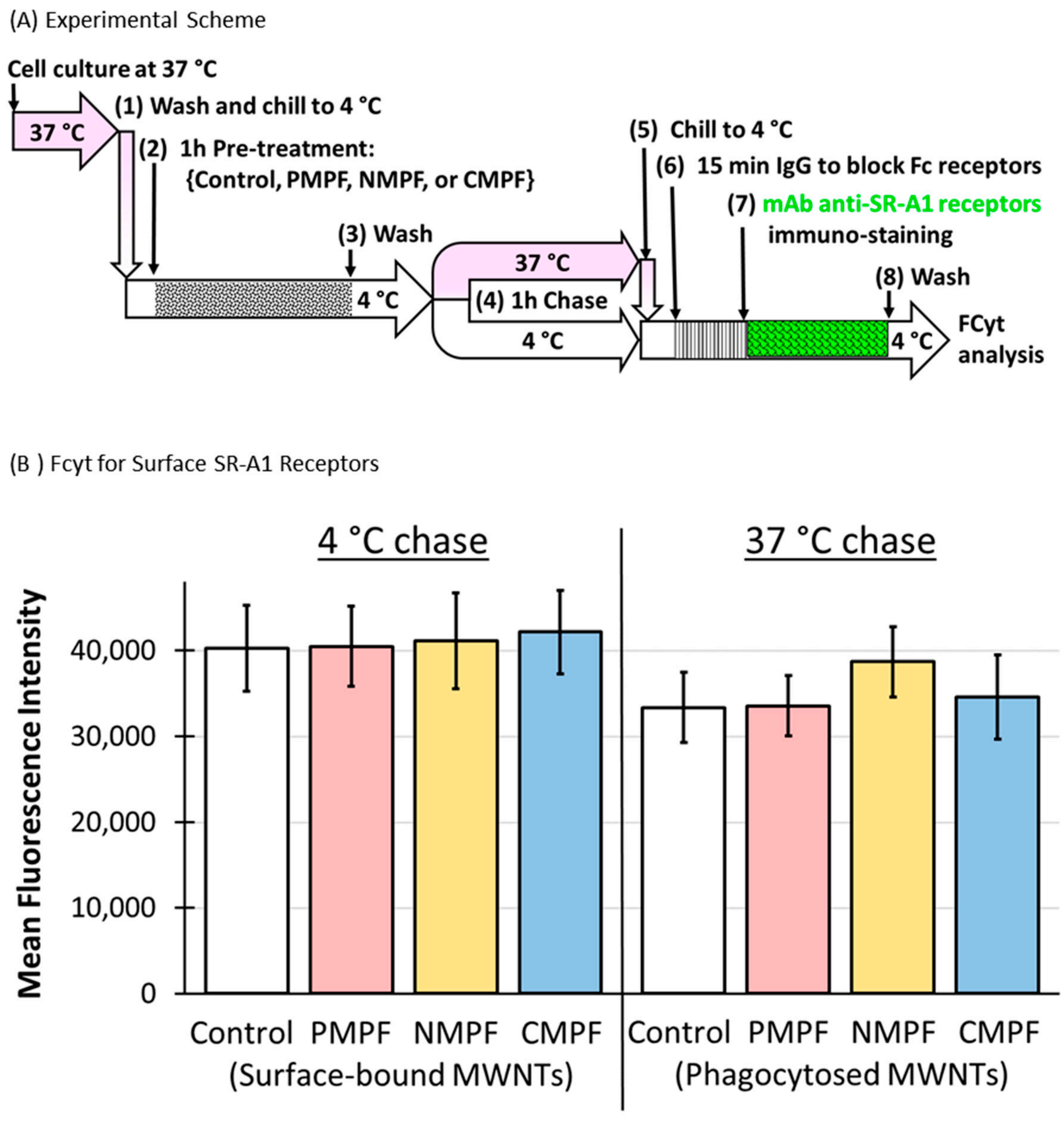

SI Figure S3. Surface-bound or phagocytosed MWNTs do not interfere with immunofluorescence FCyt assays for surface SR-A1 receptors on RAW 264.7 cells. RAW 264.7 cells were incubated in media containing 100 μg/mL of P-, N-, or C-MWNTs at 4 °C for 1 h to allow binding of MWNTs to the cell surface and then washed to remove unbound MWNTs.

Control cells were kept in medium in the absence of MWNTs at 4 °C. Half of the cells were chased at 37 °C in fresh medium to allow phagocytosis of MWNTs, the other half were kept at 4 °C. To detect SR-A1 receptors on the cell surface, cells were detached from culture plates with enzyme-free buffer, washed, and chilled on ice. Surface Fc receptors were blocked with mouse IgG. For immunostaining, Alexa Fluor® 488-conjugated monoclonal rat anti-mouse mSR-A1/MSR antibody specific for SR-A1 receptor or Alexa Fluor® 488-conjugated monoclonal rat IgG2b isotype control antibody was added and incubated with the cells for 30 min in the dark. Cells were washed to remove unbound antibodies prior to assessment for the presence of surface SR-A1 receptors by FCyt. (A) The time-line scheme outlines the key experimental steps. The mean fluorescence intensity value was determined from a total of 20,000 cells analyzed per measurement. (B) The surface SR-A1 receptors expression levels of cells kept at 4 °C (left side) and cells chased at 37 °C (right side) are represented by their mean fluorescence intensities. Data is the mean ± SD of triplicate measurements per sample in ≥3 independent experiments.

SI Figure S4

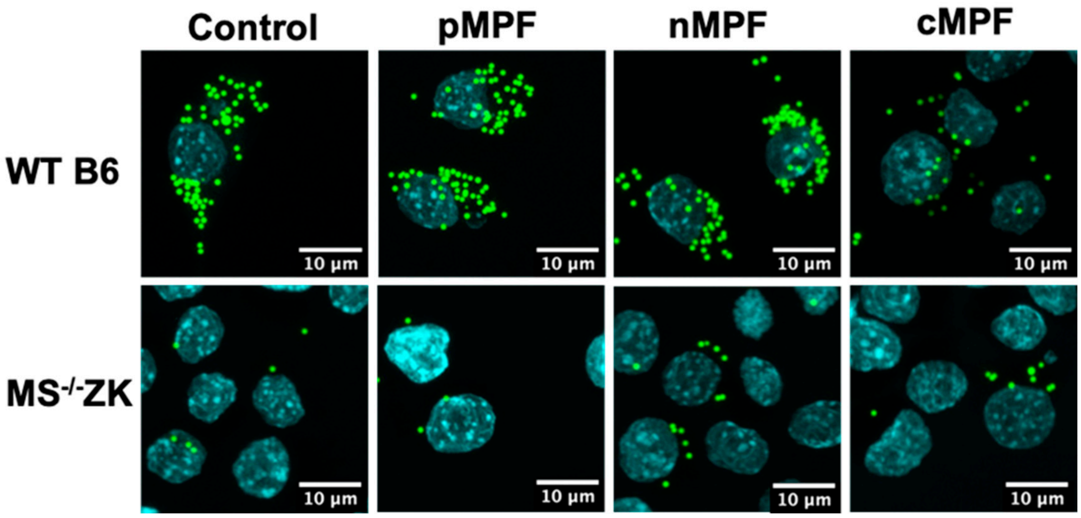

SI Figure S4. Effects of MWNT accumulation on subsequent phagocytosis of polystyrene beads in WT B6 and MS$^{-/-}$ ZK cells assessed by LSCFM. See Figure 6A for a time-line scheme that outlines the key experimental steps in the protocol. Wild type B6 and MS$^{-/-}$ ZK cells were grown on glass cover slips and incubated in media containing 100 µg/mL of P-, N-, or C-MWNTs at 37 °C for 20 h. Untreated control cells were incubated in regular culture medium in the absence of MWNTs. Cells were washed and incubated in fresh medium at 37 °C for 1 h to promote internalization of MWNTs from the cell surface. Cells were exposed to 25 µg/mL non-functionalized, fluorescent-conjugated polystyrene beads (1 µm in diameter) in fresh medium at 37 °C for 2 h. Cells were washed to remove excess polystyrene beads in the medium, chased in fresh medium for 1 h, incubated with Hoechst 33342 dye, fixed with 4% PFA, and mounted on glass slides. Representative LSCFM images of WT B6 (top row) and MS$^{-/-}$ ZK (bottom row) control and cells pre-treated with 100 µg/mL of P-, N-, or C-MWNTs were acquired using a laser scanning confocal fluorescence microscope with a 100x objective lens magnification, as described in Methods. Blue fluorescence is emitted from Hoechst 33342 stained nuclei and green fluorescence is from phagocytosed polystyrene beads.

SI Figure S5.

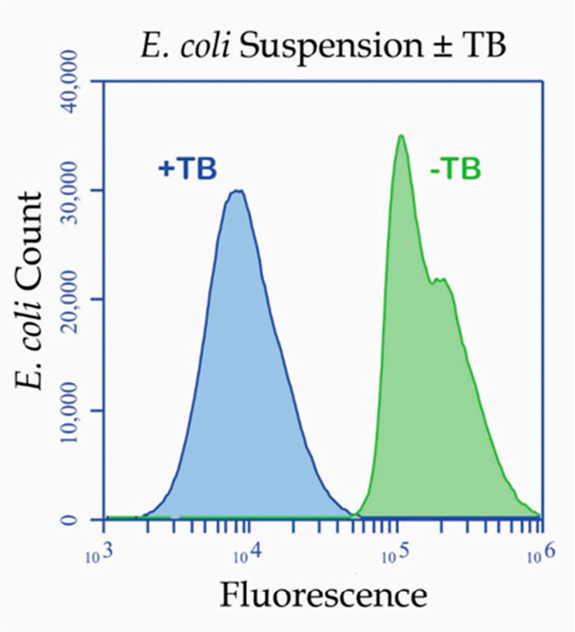

Sample	TB	Counts	Mean Fluorescence Intensity	Quenching
E. coli Stock	-	1,000,000	197,976 ± 3,515	94.4%
	+	1,000,000	11,054 ± 277	

SI Figure S5. Fluorescence quenching of fluorescent-conjugated, heat killed *E. coli* particles by trypan blue dye. As received heat-killed *E. coli* (K-12 strain) BioParticles® covalently conjugated with Alexa Fluor® 488 (Invitrogen Cat. No. E13231) were reconstituted in PBS with 2 mM sodium azide. Aliquots of *E. coli* particle stock suspension with aggregates were sonicated using an ultrasonic bath sonicator (Elmasonic P30H), operated at 37 kHz, 120 W, for 2 × 2 min at 4 °C. The *E. coli* particle suspension appeared homogenous and lacked visible aggregates upon inspection using a light microscope with a 40× objective lens. The *E. coli* were diluted in PBS and the titer was calculated from triplicated measurements where the volume that contains 1×10^6 counts was recorded using a flow cytometer. The mean fluorescence intensity was acquired before

(-TB) and after (+TB) 0.1% trypan blue was added. The magnitude of fluorescence quenched by trypan blue is defined as the difference in fluorescence intensities measured in the absence and presence of trypan blue dye. The percent quenching by trypan blue was calculated as {(-TB) – (+TB)}/(-TB), where the intensity in the absence of dye was set to 100%. Data is the mean ± SD of triplicate measurements per sample.

SI Figure S6.

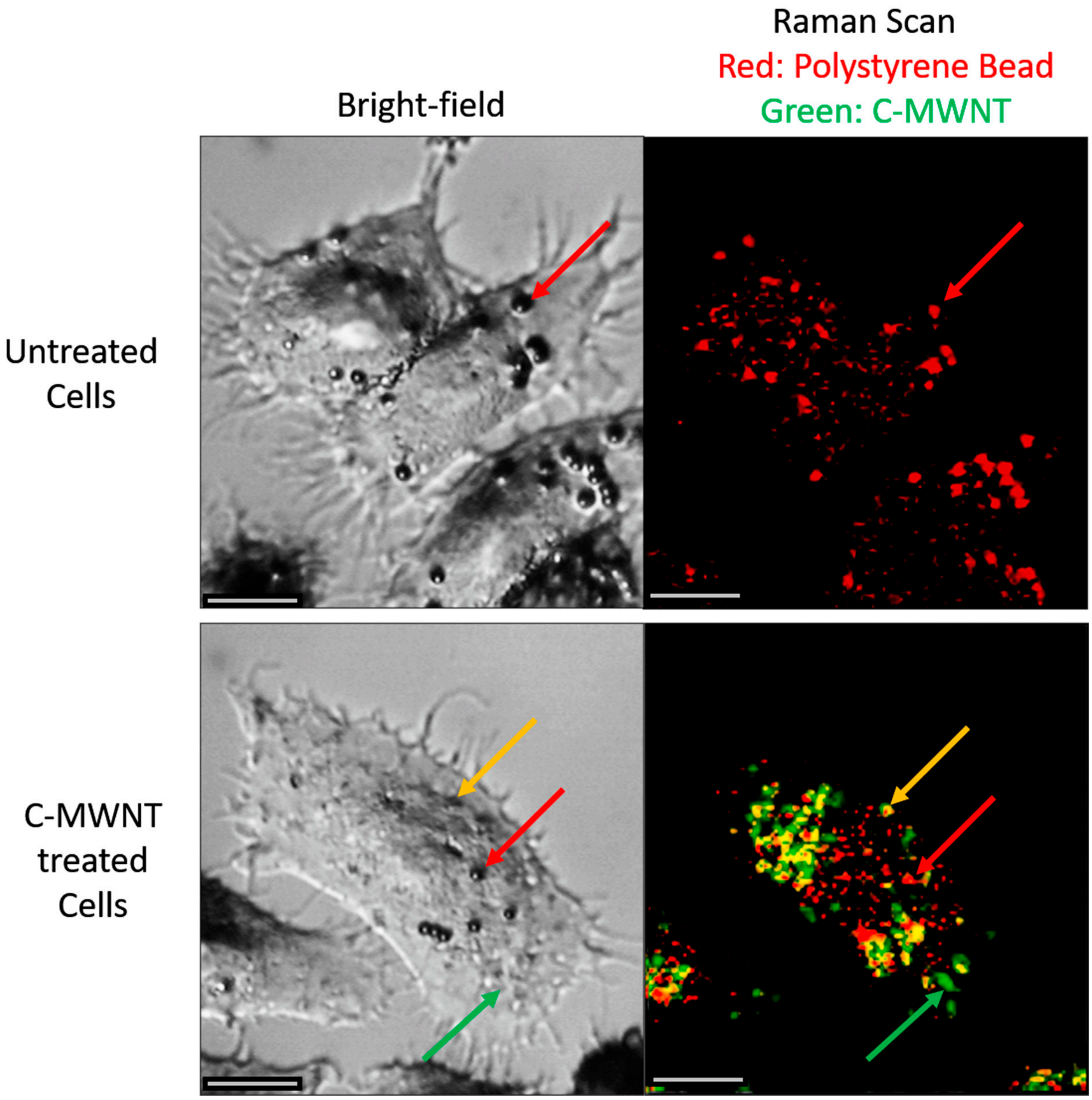

SI Figure S6. Laser scanning confocal Raman microscopy analysis of RAW 264.7 cells with phagocytosed polystyrene beads in the present or absence of C-MWNTs. Raw 264.7 cells cultured on glass coverslips were incubated in regular culture medium as control or in test medium contained PF108-coated C-MWNTs at 100 µg/mL for 2 h at 37 °C. After the pre-treatment, cells were washed and exposed to non-fluorescent and non-functionalized 0.9 µm polystyrene beads at 25 µg/mL for 2 h at 37 °C, washed thoroughly again, fixed with 4% PFA, and then air-dried. Laser

scanning confocal Raman images of the cells were acquired using a *WITec 500R* laser scanning confocal Raman microscopy system equipped with a 532 nm laser and a 100× objective lens, as described in Supplement Methods. The scale bars are 10 μm.

Top row: A representative image of control cells exposed only to polystyrene beads for 2 h at 37 °C. The bright-field image on the top-left panel shows bead-like structures in the perinuclear region characteristic of phagolysosomes. The Raman scan image on the top-right panel shows strong polystyrene-specific Raman signals detected and colored in red, showing the location of the beads. The red arrow points to the area with Raman signal from one bead.

Bottom row: A representative image of cells first exposed for 2h at 37 °C to C-MWNT, washed, then exposed to polystyrene beads for 2 h at 37 °C. Similar to the control cells on the top-left panel, the bright-field image on the bottom-left panel shows beads in the C-MWNT treated cells. The Raman image on the bottom-right panel shows the polystyrene-specific Raman signal colored in red overlaid with the MWNT-specific Raman signal colored in green. Pixels with visible yellow result when C-MWNTs and beads are co-localized, such as would occur if both C-MWNTs and beads were in the same phagolysosomes. The yellow arrow identifies one area of several in the field where green and red overlap giving yellow, indicating colocalization of bead and C-MWNTs. The green arrow identifies an area with strong MWNT signals colored in green. The red arrow points to an area with strong red polystyrene signals in red, but no green or yellow, suggesting the presence of a bead but not C-MWNTs at this location.

SI Figure S7.

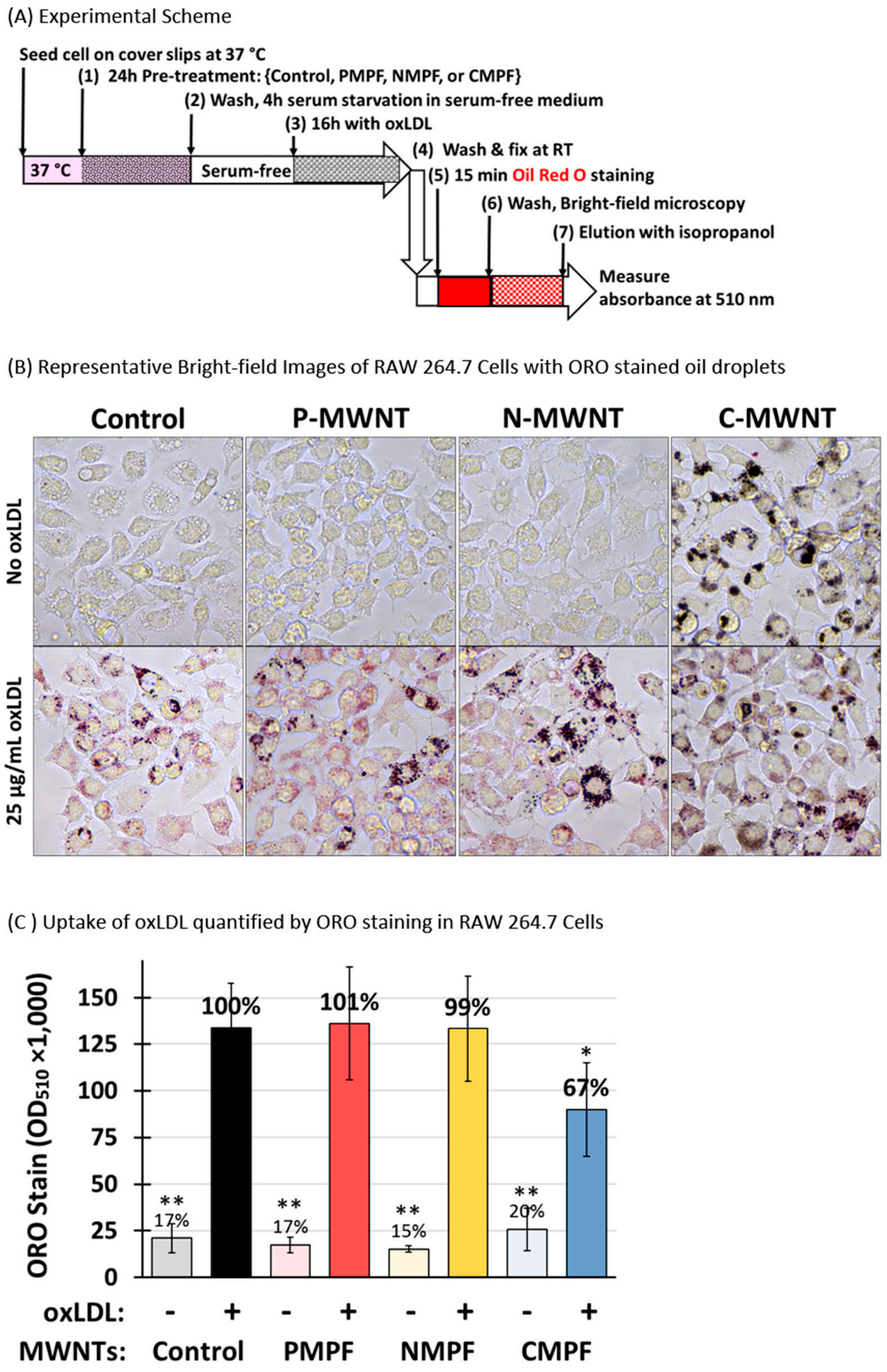
(A) Experimental Scheme
Seed cell on cover slips at 37 °C
(1) 24h Pre-treatment: {Control, PMPF, NMPF, or CMPF}
(2) Wash, 4h serum starvation in serum-free medium
(3) 16h with oxLDL
(4) Wash & fix at RT
(5) 15 min Oil Red O staining
(6) Wash, Bright-field microscopy
(7) Elution with isopropanol
37 °C
Serum-free
Measure absorbance at 510 nm
(B) Representative Bright-field Images of RAW 264.7 Cells with ORO stained oil droplets
Control
P-MWNT
N-MWNT
C-MWNT
No oxLDL
25 µg/mL oxLDL
(C) Uptake of oxLDL quantified by ORO staining in RAW 264.7 Cells
ORO Stain (OD510 ×1,000)
0
25
50
75
100
125
150
** 17%
100%
** 17%
101%
** 15%
99%
** 20%
* 67%
oxLDL: - + - + - + - +
MWNTs: Control PMPF NMPF CMPF

SI Figure S7. Reduced oxLDL uptake by RAW 264.7 cells pre-treated with C-MWNTs, but not P- or N-MWNTs, assessed using ORO staining assays. RAW 264.7 cells were seeded on glass coverslips and incubated in media containing 100 µg/mL of P-, N-, or C-MWNTs at 37 °C for 24 h. Untreated control cells were incubated in regular culture medium in the absence of PF108 surfactant or MWNTs. After the pre-treatments, cells were washed and serum starved for 4 h in serum-free medium supplemented with 3% *w/v* BSA (SF+BSA medium) at 37 °C before incubation in fresh SF+BSA medium in the presence or absence of 25 µg/mL oxLDL for 16 h at 37 °C. Cells were washed again, fixed with 4% PFA, and oil droplets in cells were stained with freshly prepared 0.3% *w/v* ORO in 60% isopropanol for 15 min at room temperature. Unbound ORO dyes were removed by extensive washing with water and the coverslips were dipped in water and 50% isopropanol carefully before being transferred to new 4-well plates to reduce background staining. Bright-field images illuminated with an LED transmitted light were acquired with a 40× magnification objective lens. The ORO dye in cells was eluted in 100% isopropanol and the eluate was transferred to a clear 96-well plate where the red dye intensity was measured at 510 nm using a microplate reader. (A) The time-line scheme outlines the key experimental steps. (B) Representative ORO stained bright-field images of untreated control and MWNT-treated cells with or without oxLDL exposure. (C) The amount of oil droplet per sample, represented as the mean ORO intensity in isopropanol eluate, was determined by the absorbance at 510 nm and plotted as bar graph. Data is the mean ± SD of duplicate samples in ≥ 3 independent experiments. Percent shown above a bar indicates mean ORO intensity relative to untreated control cells incubated with oxLDL where the level of OD_{510} was set to 100%. * is for $P < 1.0 \times 10^{-2}$ and ** is for $P < 1.0 \times 10^{-4}$ against control with oxLDL uptake.
