# Supplementary material for: Selective Uptake of Carboxylated Multi-Walled Carbon Nanotubes by Class A Type 1 Scavenger Receptors and Impaired Phagocytosis in Alveolar Macrophages
Source: Nanomaterials (Basel). 2020 Dec 3;10(12):2417. doi: 10.3390/nano10122417 (PMC7761752; doi:10.3390/nano10122417)
Supplement: Supplementary file 1 [file nanomaterials-10-02417-s001.zip › nanomaterials-988389-update SI 2/UPDATE - ACCESSING VIDEO FILES IN FIGURE S2.pdf]

UPDATE: ACCESSING VIDEO FILES IN FIGURE S2

Supplemental Figure S2 instructs readers to open the videos in panels A and B by clicking the panel. However, this link does not work. Now, videos in Figure S2 A and B are uploaded as standalone files in the Supplemental Material and can be directly opened by clicking on them.
